# Jumper enables discontinuous transcript assembly in coronaviruses

**DOI:** 10.1038/s41467-021-26944-y

**Published:** 2021-11-18

**Authors:** Palash Sashittal, Chuanyi Zhang, Jian Peng, Mohammed El-Kebir

**Affiliations:** 1 Department of Computer Science, University of Illinois at Urbana-Champaign, Urbana, IL 61801 USA; 2 Department of Electrical and Computer Engineering, University of Illinois at Urbana-Champaign, Urbana, IL 61801 USA; 3 College of Medicine, University of ILlinois at Urbana-Champaign, Urbana, IL 61801 USA

**Keywords:** Software, Gene expression, RNA splicing, Genome informatics

## Abstract

Genes in SARS-CoV-2 and other viruses in the order of *Nidovirales* are expressed by a process of discontinuous transcription which is distinct from alternative splicing in eukaryotes and is mediated by the viral RNA-dependent RNA polymerase. Here, we introduce the DISCONTINUOUS TRANSCRIPT ASSEMBLYproblem of finding transcripts and their abundances given an alignment of paired-end short reads under a maximum likelihood model that accounts for varying transcript lengths. We show, using simulations, that our method, JUMPER, outperforms existing methods for classical transcript assembly. On short-read data of SARS-CoV-1, SARS-CoV-2 and MERS-CoV samples, we find that JUMPER not only identifies canonical transcripts that are part of the reference transcriptome, but also predicts expression of non-canonical transcripts that are supported by subsequent orthogonal analyses. Moreover, application of JUMPER on samples with and without treatment reveals viral drug response at the transcript level. As such, JUMPER enables detailed analyses of *Nidovirales* transcriptomes under varying conditions.

## Abstract (web summary)

@melkebir @psashittal et al. develop a graph-based method for the assembly of discontinuous transcripts produced in Coronaviruses and other Nidovirales, enabling the discovery of transcriptional changes missed by existing methods.

## Introduction

**Fig. 1:**
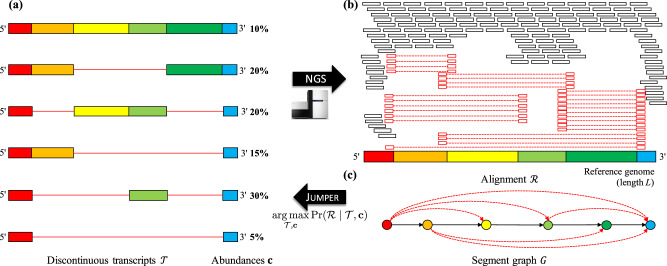
Overview of Jumper. **a** Coronaviruses generate a set ${{{{{{{\mathcal{T}}}}}}}}$ of discontinuous transcripts with varying abundances (**c**) during infection. **b** Next-generation sequencing will produce an alignment ${{{{{{{\mathcal{R}}}}}}}}$ with two types of aligned reads: reads that map to a contiguous genomic region (black) and split reads that map to distinct genomic regions (red). **c** From ${{{{{{{\mathcal{R}}}}}}}}$ we obtain the segment graph *G*, a directed acyclic graph with a unique Hamiltonian path. JUMPER solves the DISCONTINUOUS TRANSCRIPT ASSEMBLYto infer ${{{{{{{\mathcal{T}}}}}}}}$ and **c** with maximum likelihood. While this figure shows single-end reads, our problem statement and method make use of the additional information provided by paired-end reads.

Coronaviruses, and more generally viruses in the taxonomic order of *Nidovirales*, are enveloped viruses containing a positive-sense, single-stranded RNA genome that encodes for non-structural proteins near the 5ʹ end as well as structural and accessory proteins near the 3ʹ end^1^. Since the host ribosome processes mRNA starting at the 5ʹ end, translation of the viral genome only generates the non-structural proteins. Expression of the remaining genes is achieved by discontinuous transcription performed by the viral RNA-dependent RNA polymerase (RdRp)^2^, a protein that is encoded in the non-structural part of the viral genome. Specifically, RdRp can skip over contiguous genomic regions, or segments, in the viral RNA template, resulting in a repertoire of discontinuous transcripts that correspond to distinct subsequences of segments ordered as in the reference genome (Fig. 1a). Several recent studies have analyzed SARS-CoV-2 sequencing samples, identifying split reads—i.e. reads that span non-contiguous parts of the viral genome—that provide evidence for canonical discontinuous transcription events that produce an intact 3ʹ open reading frame as well as non-canonical discontinuous transcription events whose role is unclear^3–5^. However, to the best of our knowledge, no study has attempted to assemble coronavirus transcriptomes, which could provide important clues about the viral life cycle under various conditions such as drug treatment.

Current methods for transcript assembly are mainly designed for eukaryotes and fall under two broad categories: (i) reference-based methods and (ii) de novo assembly methods. The main distinction is that the former require the reference genome as input while the latter have no such requirement. As such, de novo assembly methods^6–10^ are useful when the reference genome is unavailable or when the diversity of different species in the sample is too large. On the other hand, reference-based methods^11–21^ generally achieve higher accuracy as they use the reference genome as a scaffold on which to align sequencing reads. Typically, such methods construct a splice graph *G*—i.e. a directed graph whose nodes correspond to contiguous genomic regions and edges indicate splice junctions—and subsequently aim to decompose the graph into paths that correspond to transcripts. In addition to inferring transcripts ${{{{{{{\mathcal{T}}}}}}}}$ given an alignment ${{{{{{{\mathcal{R}}}}}}}}$, a subset of reference-based methods simultaneously estimates their abundances **c**^17,19^. Alternatively, transcripts abundances **c** may also be quantified using separate tools^22,23^. We refer to Supplementary Note A.1 for a more detailed overview of previous work.

There are important differences between transcription in eukaryotes and coronaviruses. In eukaryotes, a gene may express multiple transcripts that differ in their composition due to alternative splicing, which is predominantly mediated by the spliceosome and results in the generation of multiple mRNAs with differentially joined or skipped exons (segments) from the same gene. By contrast, transcripts in coronaviruses result from discontinuous transcription, which is mediated by viral RdRp and results in the removal of contiguous segments due to jumps of the RdRp. There are two key differences between these two biological processes. First, in discontinuous transcription, there is no shuffling of segments and the ordering of the segments is maintained in each transcript. Since exon shuffling in eukaryotes is rare during alternative splicing, this constraint is commonly used in existing transcript assembly methods as well^11,12,19–21^. Second, the complete viral genome, without any jumps, is always part of the transcriptome. Consequently, due to these two biological constraints, an alignment ${{{{{{{\mathcal{R}}}}}}}}$ of coronavirus samples will yield a splice graph *G* with additional constraints that current methods do not leverage.

In this study, we introduce the DISCONTINUOUS TRANSCRIPT ASSEMBLY (DTA) problem of finding discontinuous transcripts ${{{{{{{\mathcal{T}}}}}}}}$ and their abundances **c** (Fig. 1a) given an alignment ${{{{{{{\mathcal{R}}}}}}}}$ of paired-end reads (Fig. 1b). Underpinning our approach is the concept of a segment graph (Fig. 1c), which is an acyclic splice graph with a Hamiltonian path due to the aforementioned constraints. This enables us to characterize discontinuous transcripts ${{{{{{{\mathcal{T}}}}}}}}$ as small subsets of non-overlapping edges in this graph. Our method, JUMPER, uses this compact representation to solve the DTA at scale via a progressive heuristic that incorporates a mixed integer linear program. Using simulations, we show that JUMPER outperforms SCALLOP^11^ and STRINGTIE^12^, existing methods for reference-based transcript assembly in eukaryotes. In real data^3^, we run JUMPER on paired-end short-read data of virus-infected Vero cells and use long-read data of the same sample for validation. We find that JUMPER not only identifies canonical transcripts that are part of the reference transcriptome, but also predicts expression of non-canonical transcripts that are well supported by long-read data. Similarly, JUMPER identifies canonical and non-canonical transcripts in SARS-CoV-1 and MERS-CoV samples^24^. Finally, we demonstrate the use of JUMPER to study viral drug response at the transcript level by analyzing samples with and without treatment prior to infection^25^. In summary, JUMPER enables detailed analyses of coronavirus transcriptomes under varying conditions.

## Results

### Discontinuous Transcript Assembly problem

To formulate the DISCONTINUOUS TRANSCRIPT ASSEMBLY problem, we define discontinuous transcripts as sequences of segments whose order matches the reference genome. More formally, we have the following definition.

#### Definition 1

Given a reference genome, a *discontinuous transcript* *T* is a sequence **v**_1_, …, **v**_∣*T*∣_ of segments where (i) each segment corresponds to a contiguous region in the reference genome, (ii) segment **v**_*i*_ precedes segment **v**_*i* + 1_ in the reference genome for all *i* ∈ {1, …, ∣*T*∣ − 1}, (iii) segment **v**_1_ contains the 5ʹ end of the reference genome and (iv) segment **v**_∣*T*∣_ contains the 3ʹ end of the reference genome.

While the genomic transcript *T*_0_ matches the reference genome^2^, subgenomic transcripts contain jumps and correspond to subgenomic RNAs (sgRNAs)^3^. Discontinuous transcripts ${{{{{{{\mathcal{T}}}}}}}}=\{{T}_{i}\}$ occur in abundances **c** = [*c*_*i*_] where *c*_*i*_ ≥ 0 is the relative abundance of transcript *T*_*i*_ such that $\mathop{\sum }\nolimits_{i = 1}^{| {{{{{{{\mathcal{T}}}}}}}}| }{c}_{i}=1$. In this work, we focus on coronavirus sequencing samples obtained using Illumina sequencing, where reads originate from the reference genome of length *L* of about 10–30 Kbp and have a fixed length *ℓ* ranging from 100 to 400 bp. We refer to Supplementary Note A.3 for a discussion on why transcript assembly remains relevant for such samples in light of the availability long-read sequencing samples. For ease of exposition, we describe the formulation in the context of single-end reads, but in practice we use the paired-end information if it is available. We refer to Supplementary Note B.5 for details on the paired-end formulation.

As *ℓ* ≪ *L*, the identity of the transcript of origin for a given read is ambiguous. Therefore, we need to use computational methods to reconstruct the transcripts and their abundances from the sequencing reads. Specifically, given a coronavirus reference genome of length *L* and reads of a fixed length *ℓ*, we use a splice-aware aligner such as STAR^26^ to obtain an alignment ${{{{{{{\mathcal{R}}}}}}}}$. This alignment provides information about the abundance **c** and composition of the underlying transcripts ${{{{{{{\mathcal{T}}}}}}}}$ in the following two ways. First, the depth, or the number of reads along the genome is informative for quantifying the abundance **c** of the transcripts. Second, the composition of the transcripts ${{{{{{{\mathcal{T}}}}}}}}$ is embedded in split reads, which are reads that align to multiple distinct regions in the reference genome (Fig. 1b). Since the alignment ${{{{{{{\mathcal{R}}}}}}}}$ is composed of reads from discontinuous transcripts, the alignment satisfies the following two properties. First, the genomic regions induced by any read in ${{{{{{{\mathcal{R}}}}}}}}$, including split reads, are ordered from the 5ʹ to 3ʹ direction of the reference genome. Second, due to the presence of the genomic transcript *T*_0_, every position in the reference genome can be expected to be covered by a read.

To infer ${{{{{{{\mathcal{T}}}}}}}}$ and **c** from ${{{{{{{\mathcal{R}}}}}}}}$, most reference-based transcript assembly methods employ a splice graph^11,12,18^. Informally, the nodes of this graph correspond to contiguous segments of the genome (i.e. are not separated by any split read) and directed edges correspond to pairs of segments that are spanned by the same read. Due to the aforementioned properties of an alignment ${{{{{{{\mathcal{R}}}}}}}}$ of reads from discontinuous transcripts, the edges of the corresponding splice graph can be partitioned into two sets. First, continuous edges correspond to edges between segments that are adjacent in the reference genome. Conversely, due to the presence of reads from the genomic transcript *T*_0_, every pair of adjacent segments in the reference genome will be connected by a continuous edge. Second, discontinuous edges connect non-adjacent segments, which indicate the jumps made by the viral RdRp during discontinuous transcription. Both types of directed edges connect segments in the same as the reference genome. Thus, the splice graph obtained from ${{{{{{{\mathcal{R}}}}}}}}$ is a directed acyclic graph (DAG) with a Hamiltonian path composed of the continuous edges.

**Fig. 2:**
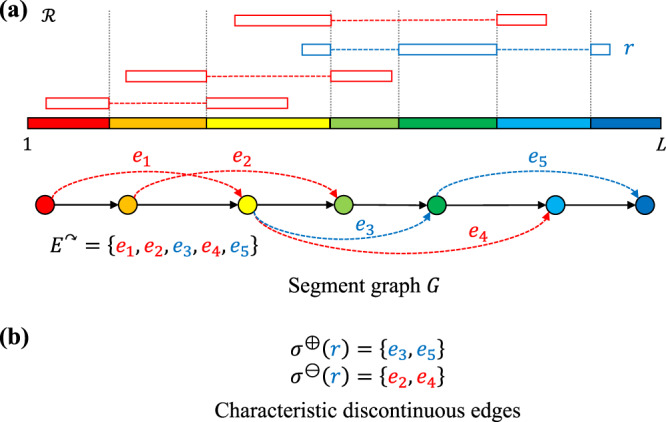
Schematic describing split reads and characteristic discontinuous edges. **a** Split reads in an alignment ${{{{{{{\mathcal{R}}}}}}}}$ define a set of junctions, which in turn define the segment graph *G*. **b** Each split read has characteristic discontinuous edges indicating the set *σ*^⊕^ of discontinuous edges present in the read as well as conflicting/overlapping discontinuous edges *σ*^⊖^. Here, split read *r* (blue), has *σ*^⊕^(*r*) = {*e*_3_, *e*_5_} and *σ*^⊖^(*r*) = {*e*_2_, *e*_4_}. Note that *e*_1_ is not included in *σ*^⊖^(*r*) as it does not overlap with *π*(*r*) = {*e*_3_, *e*_5_}.

We now show an alternative, more efficient construction of the same graph using only the split reads in an alignment ${{{{{{{\mathcal{R}}}}}}}}$ of reads from discontinuous transcripts. Each split read $r\in {{{{{{{\mathcal{R}}}}}}}}$ maps to *q* ≥ 2 distinct regions in the reference genome. Each pair of regions that are adjacent in the split read are separated by two positions *v*, *w* (where *w* − *v* ≥ 2) in the reference genome called junctions. Thus, each split read contributes 2*q* − 2 junctions. The collective set of junctions contributed by all split reads in ${{{{{{{\mathcal{R}}}}}}}}$ in combination with positions {1, *L*} induces a partition of the reference genome into closed intervals [*v*^−^, *v*^+^] of junctions that are consecutive in the reference genome (i.e. there exists no other junction that occurs in between *v*^−^ and *v*^+^). The resulting set of segments equals the node set *V* of segment graph *G* (Fig. 2a). The edge set *E* of segment graph *G* is composed of continuous edges *E*^→^ and discontinuous edges *E*^↷^. Continuous edges *E*^→^ are composed of ordered pairs (**v** = [*v*^−^, *v*^+^], **w** = [*w*^−^, *w*^+^]) of nodes that correspond to segments that are adjacent in the reference genome, i.e. where *v*^+^ = *w*^−^. On the other hand, discontinuous edges *E*^↷^ are composed of ordered pairs (**v** = [*v*^−^, *v*^+^], **w** = [*w*^−^, *w*^+^]) of nodes that corresponds to segments that are adjacent in at least one split read in ${{{{{{{\mathcal{R}}}}}}}}$ but not adjacent in the reference genome (i.e. *w*^−^ − *v*^+^ ≥ 2). Fig. 1c shows an example of a segment graph.

#### Definition 2

Given an alignment ${{{{{{{\mathcal{R}}}}}}}}$ of reads from discontinuous transcripts, the corresponding *segment graph* *G* = (*V*, *E*^→^ ∪ *E*^↷^) is a directed graph whose node set *V* equals the set of segments induced by the junctions of split reads in ${{{{{{{\mathcal{R}}}}}}}}$ and whose edge set *E* = *E*^→^ ∪ *E*^↷^ is composed of edges (**v** = [*v*^−^, *v*^+^], **w** = [*w*^−^, *w*^+^]) that are either continuous, i.e. *v*^+^ = *w*^−^, or discontinuous, i.e. *w*^−^ − *v*^+^ ≥ 2 and there exists a split read where junctions *v*^+^ and *w*^−^ are adjacent.

Thus, by definition any segment graph will have a Hamiltonian path induced by the continuous edges *E*^→^. Moreover, the segment graph obtained from an alignment of reads from discontinuous transcripts will be a DAG.

#### Observation 1

The segment graph *G* obtained from an alignment of reads from discontinuous transcripts is a directed acyclic graph with a (unique) Hamiltonian path.

By the above observation, *G* has a unique source node **s** and sink node **t**. Importantly, each transcript $T\in {{{{{{{\mathcal{T}}}}}}}}$ that is compatible with an alignment ${{{{{{{\mathcal{R}}}}}}}}$ corresponds to an **s** − **t** path *π*(*T*) in *G*. Here, a path *π* is a subset of edges *E* that can be ordered (**v**_1_, **w**_1_), …, (**v**_∣*π*∣_, **w**_∣*π*∣_) such that **w**_*i*_ = **v**_*i*+1_ for all *i* ∈ [∣*π*∣ − 1] = {1, …, ∣*π*∣ − 1}. While splice graphs of general alignments are DAGs and typically have a unique source and sink node as well, they do not necessarily contain a Hamiltonian path^11,19,27,28^.

Our goal is to find a set ${{{{{{{\mathcal{T}}}}}}}}$ of transcripts and their abundances **c** that maximize the posterior probability

$$\Pr ({{{{{{{\mathcal{T}}}}}}}},{{{{{{{\bf{c}}}}}}}}| {{{{{{{\mathcal{R}}}}}}}})\propto \Pr ({{{{{{{\mathcal{R}}}}}}}}| {{{{{{{\mathcal{T}}}}}}}},{{{{{{{\bf{c}}}}}}}})\Pr ({{{{{{{\mathcal{T}}}}}}}},{{{{{{{\bf{c}}}}}}}}).$$

Under an uninformative, flat prior $\Pr ({{{{{{{\mathcal{T}}}}}}}},{{{{{{{\bf{c}}}}}}}})$, this is equivalent to maximizing the probability $\Pr ({{{{{{{\mathcal{R}}}}}}}}| {{{{{{{\mathcal{T}}}}}}}},{{{{{{{\bf{c}}}}}}}})$. We use the segment graph *G* to compute the probability $\Pr ({{{{{{{\mathcal{R}}}}}}}}| {{{{{{{\mathcal{T}}}}}}}},{{{{{{{\bf{c}}}}}}}})$ of observing an alignment ${{{{{{{\mathcal{R}}}}}}}}$ given transcripts ${{{{{{{\mathcal{T}}}}}}}}$ and abundances **c**. We follow the same generative model which has been extensively used for transcription quantification^22,23,29^. The notations used in this paper best resemble the formulation described in ref. ^28^. Let ${{{{{{{\mathcal{R}}}}}}}}$ be composed of reads {*r*_1_, …, *r*_*n*_} and the set ${{{{{{{\mathcal{T}}}}}}}}$ of transcripts be {*T*_1_, …, *T*_*k*_} with lengths *L*_1_, …, *L*_*k*_ and abundances **c** = [*c*_1_, …, *c*_*k*_]. In line with current literature, reads ${{{{{{{\mathcal{R}}}}}}}}$ are generated independently from transcripts ${{{{{{{\mathcal{T}}}}}}}}$ with abundances **c**. Further, we must marginalize over the set of transcripts ${{{{{{{\mathcal{T}}}}}}}}$ as the transcript of origin of any given read is typically unknown, since *ℓ* ≪ *L*. Moreover, we assume that the fixed read length *ℓ* is much smaller than the length *L*_*i*_ of any transcript *T*_*i*_. As such, we have that $\Pr ({{{{{{{\mathcal{R}}}}}}}}| {{{{{{{\mathcal{T}}}}}}}},{{{{{{{\bf{c}}}}}}}})$ equals

1 $$\Pr ({{{{{{{\mathcal{R}}}}}}}}| {{{{{{{\mathcal{T}}}}}}}},{{{{{{{\bf{c}}}}}}}}) =	\mathop{\prod }\limits_{j=1}^{n}\Pr ({r}_{j}| {{{{{{{\mathcal{T}}}}}}}},{{{{{{{\bf{c}}}}}}}})\\ =	\mathop{\prod }\limits_{j = 1}^{n}\frac{1}{\mathop{\sum }\nolimits_{b=1}^{k}{c}_{b}{L}_{b}}\mathop{\sum}\limits_{i:\pi ({T}_{i})\supseteq \pi ({r}_{j})}{c}_{i},$$

where *π*(*T*) ⊆ *E* is the **s** − **t** path corresponding to transcript *T* and *π*(*r*) ⊆ *E* is the path induced by the ordered sequence of segments (or nodes of *G*) spanned by read *r*. By construction, *π*(*T*) ⊇ *π*(*r*) is a necessary condition for transcript *T* to be a candidate transcript of origin of read *r*. Supplementary Note A.2 gives the derivation of the above equation (Eq. (1)). Our goal is to find ${{{{{{{\mathrm{argmax}}}}}}}\,}_{{{{{{{{\mathcal{T}}}}}}}},{{{{{{{\bf{c}}}}}}}}}\Pr ({{{{{{{\mathcal{R}}}}}}}}| {{{{{{{\mathcal{T}}}}}}}},{{{{{{{\bf{c}}}}}}}})$, leading to the following problem.

#### Problem 1

(DISCONTINUOUS TRANSCRIPT ASSEMBLY(DTA)). Given alignment ${{{{{{{\mathcal{R}}}}}}}}$ and integer *k*, find discontinuous transcripts ${{{{{{{\mathcal{T}}}}}}}}=\{{T}_{1},\ldots ,{T}_{k}\}$ and abundances **c** = [*c*_1_, …, *c*_*k*_] such that (i) each transcript ${T}_{i}\in {{{{{{{\mathcal{T}}}}}}}}$ is an **s** − **t** path in segment graph *G*, and (ii) $\Pr ({{{{{{{\mathcal{R}}}}}}}}| {{{{{{{\mathcal{T}}}}}}}},{{{{{{{\bf{c}}}}}}}})$ is maximum.

In practice, we set the value of *k* to a large number (e.g. *k* = 50) and restrict the subsequent analyses to the set of transcripts whose abundance exceeds a threshold value (e.g. ≥0.001). The probability $P({{{{{{{\mathcal{R}}}}}}}}| {{{{{{{\mathcal{T}}}}}}}},{{{{{{{\bf{c}}}}}}}})$, in Eq. (1), is expressed in terms of the observed reads and their induced paths *π*(*r*) ⊆ *E*(*G*) in the segment graph *G*. In the ‘Methods’ section, we describe a more concise way of expressing the probability $P({{{{{{{\mathcal{R}}}}}}}}| {{{{{{{\mathcal{T}}}}}}}},{{{{{{{\bf{c}}}}}}}})$ using the fact that the segment graph *G* is a DAG with a unique Hamiltonian path. This concise characterization enables us to design a progressive heuristic that incorporates an efficient mixed linear integer program (MILP) to solve the DTA problem (details are in the ‘Methods’ section). Our resulting method, JUMPER, is implemented in Python 3 using Gurobi^30^ (version 9.0.3) to solve the MILP and pysam^31^ for reading and processing the input BAM file. JUMPER is available at https://github.com/elkebir-group/Jumper.

### Experimental evaluation

We begin by establishing terminology that will be used in the rest of the section. A discontinuous edge (**v** = [*v*^−^, *v*^+^], **w** = [*w*^−^, *w*^+^]) is canonical provided its 5ʹ junction *v*^+^ occurs in the transcription regulating leader sequence (TRS-L), i.e. between positions 50 and 85, and the first occurrence of ‘AUG’ downstream of the 3ʹ junction *w*^−^ position coincides with the start codon of a known open reading frame (ORF), otherwise the discontinuous edge is called non-canonical. Note that the range 50−85 is chosen since it contains the TRS-L regions of the SARS-CoV-1^32^, SARS-CoV-2^3^ and MERS-CoV^32^ genomes analyzed in this paper. In a similar vein, a transcript is canonical if it contains at most one canonical and no non-canonical discontinuous edges, otherwise the transcript is non-canonical. We ran all experiments on a server with two 2.6 GHz CPUs and 512 GB of RAM.

### Simulations

**Fig. 3:**
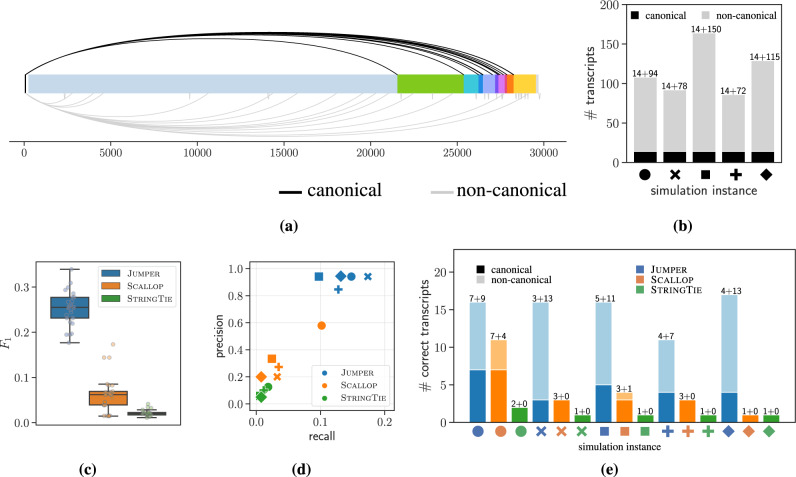
JUMPER consistently outperforms SCALLOP^11^ and STRINGTIE^12^ in reconstruction of viral transcripts from simulated SARS-CoV-2 sequencing data. **a** Sashimi plot showing the canonical (black) and non-canonical (gray) discontinuous mappings supported by reads in short-read sample SRR11409417. **b** Number of canonical and non-canonical transcripts for five simulation instances of $({{{{{{{\mathcal{T}}}}}}}},{{{{{{{\bf{c}}}}}}}})$ generated under the negative-sense discontinuous transcription model. **c** *F*_1_ score of the three methods (JUMPER, SCALLOP, and STRINGTIE) for all the 5 × 5 = 25 simulated instances (i.e. five technical replicates for each of the five simulated transcriptomes) under the negative-sense discontinuous transcription model. Box plots show the median and the interquartile range (IQR), and the whiskers denote the lowest and highest values within 1.5 times the IQR from the first and third quartiles, respectively. **d** Precision and recall values of the three methods with one of sequencing experiment for each simulated instance of $({{{{{{{\mathcal{T}}}}}}}},{{{{{{{\bf{c}}}}}}}})$ under the negative-sense discontinuous transcription model as input. **e** Total number of canonical and non-canonical transcripts recalled by the three methods for the simulated instances shown in panel (**d**).

We generated our simulation instances using a segment graph *G* obtained from a short-read sample (SRR11409417). Following Kim et al.^3^, we used fastp to trim short reads (trimming parameter set to 10 nucleotides), which were input to STAR run in two-pass mode yielding an alignment ${{{{{{{\mathcal{R}}}}}}}}$. Figure 3a shows the sashimi plot of the canonical and the non-canonical discontinuous edges (mappings) supported by the reads in the sample. From ${{{{{{{\mathcal{R}}}}}}}}$, we obtained *G* by only including discontinuous edges supported by at least 20 reads. The segment graph *G* has ∣*V*∣ = 39 nodes and ∣*E*∣ = 67 edges, which include ∣*E*^↷^∣ = 29 discontinuous edges and ∣*E*^→^∣ = 38 continuous edges. The discontinuous edges are subdivided into 14 canonical discontinuous edges that produce a known ORF and 15 non-canonical discontinuous edges. Next, we generated transcripts ${{{{{{{\mathcal{T}}}}}}}}$ and their abundances **c** from *G* using the negative-sense discontinuous transcription model (described in Supplementary Note C.1). Upon generating the transcripts, we simulated the generation and sequencing of RNA-seq data, and aligned the simulated reads using STAR^26^. We generated five independent pairs $({{{{{{{\mathcal{T}}}}}}}},{{{{{{{\bf{c}}}}}}}})$ of transcripts and abundances (Fig. 3b). For each pair $({{{{{{{\mathcal{T}}}}}}}},{{{{{{{\bf{c}}}}}}}})$ we generated five paired-end short-read sequencing simulations using polyester^33^. Thus, in total we generated 5 × 5 = 25 simulation instances.

We compare the performance of our method JUMPER with two other reference-based transcript assembly methods, SCALLOP and STRINGTIE. Note that our method, JUMPER, does not use prior knowledge about the underlying negative-sense discontinuous transcription model to infer the viral transcripts from the simulated data. To avoid including false-positive discontinuous edges, we set Λ = 100 so that JUMPER discards discontinuous edges with fewer than 100 supporting reads. For SCALLOP and STRINGTIE, we performed a sweep on their input parameters and report the best results here. We begin by comparing the transcripts predicted by the three methods to the ground-truth transcripts. Specifically, a predicted transcript is correct if there exists a transcript in the ground truth whose junction positions match the predicted junctions positions within a tolerance of ten nucleotides.

Figure 3c shows the *F*_1_ score (harmonic mean of recall and precision) of the three methods for all the simulation instances, showing that JUMPER achieves a higher *F*_1_ score (median of 0.255 and range [0.176, 0.339]) compared to SCALLOP (median of 0.062 and range [0.0145, 0.173]) and STRINGTIE (median of 0.019 and range [0.0114, 0.0412]). Supplementary Fig. 5 shows that JUMPER’s improved performance holds for both the recall and the precision with running times comparable to the SCALLOP and STRINGTIE. To investigate the effect of threshold parameter Λ on the performance of JUMPER, we ran our method on the simulated instances with Λ ∈ {10, 50, 100, 200}. Supplementary Fig. 6 shows that JUMPER outperforms SCALLOP and STRINGTIE for all values of Λ, although it incurs significantly more runtime for Λ = 10.

To better understand the tradeoff between precision and recall, we zoom in on five simulation instances with distinct pairs $({{{{{{{\mathcal{T}}}}}}}},{{{{{{{\bf{c}}}}}}}})$. Figure 3d shows the precision and recall achieved by each method for each of these five simulation instances, demonstrating that JUMPER consistently outperforms both SCALLOP and STRINGTIE. On average, JUMPER recalls 5 times more transcripts than SCALLOP and 11 times more transcripts than STRINGTIE while also having higher precision in all simulated cases. Supplementary Fig. 7 shows that all three methods produce similar precision and recall values for different sequencing replicates of the same simulated instance of $({{{{{{{\mathcal{T}}}}}}}},{{{{{{{\bf{c}}}}}}}})$, demonstrating consistency in results. Figure 3e shows the number of canonical and non-canonical transcripts generated by the three methods that match the ground truth for each simulated instance, with JUMPER consistently recalling a larger number of ground-truth canonical and non-canonical transcripts. To assess the accuracy of JUMPER’s estimation of the abundances **c**, we computed the Pearson correlation between the abundances of the correctly recalled transcripts and their ground-truth abundances. We find that JUMPER achieves a median Pearson correlation of 0.979, and that the use of SALMON to re-estimate abundances improves the median correlation to 0.985 (Supplementary Fig. 8).

In summary, we found that JUMPER correctly predicts higher number of both canonical and non-canonical transcripts compared to SCALLOP and STRINGTIE for all the simulated instances (summarized in Supplementary Table 3). We observe similar trends on simulated instances of a human gene (see Supplementary Note C.4).

### Viral transcript assembly in SARS-CoV-2-infected Vero cells

Recently, Kim et al.^3^ explored the transcriptomic architecture of SARS-CoV-2 by performing short-read as well as long-read sequencing of Vero cells infected by the virus. The authors used oligo(dT) amplification, which targets the poly(A) tail at the 3ʹ end of messenger RNAs, thus limiting positional bias that would occur when using SARS-CoV-2-specific primers^34,35^. Subsequently, the authors aligned the resulting reads using splice-aware aligners, STAR^26^ for the short-read sample (median depth of 1763) and minimap2^36^ for the long-read sample (median depth of 6707 and mean length of 2875 bp). For both complementary sequencing techniques, the authors observed split reads that were indicative of canonical as well as non-canonical transcription events. While this previous work quantified the fraction of split reads supporting each discontinuous transcription event, it did not attempt to assemble complete viral transcripts.

**Fig. 4:**
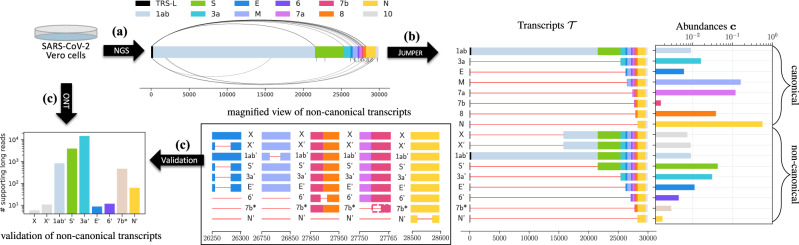
Using short-read data of SARS-CoV-2-infected Vero cells^3^, JUMPER identifies canonical and non-canonical transcripts that are well supported by long-read sequences of the same sample. **a** The segment graph for the short-read data contains both canonical (above) and non-canonical (below) edges. **b** JUMPER assembles eight canonical transcripts and nine non-canonical transcripts and estimates their abundances with zoomed-in view of the non-canonical transcripts X, X', 1ab', S', 3a', E', 6', 7b*, and N'. **c** All non-canonical transcripts predicted by JUMPER are well supported by long-read data. NGS next-generation sequencing, ONT Oxford Nanopore Technologies.

We used JUMPER to reconstruct the SARS-CoV-2 transcriptome of the short-read sequencing sample using the BAM file obtained by running Kim et al.’s pipeline^3^. This was followed by running SALMON to identify precise transcript abundances. We note that running SCALLOP on the short-read data resulted in only a single, complete canonical transcript (corresponding to ‘N’) but required subsampling of the BAM file (to 20%) due to memory constraints, whereas STRINGTIE produced two incomplete transcripts (‘ORF3a’ and a non-canonical transcript with low support). On a segment graph with ∣*V*∣ = 59 nodes and ∣*E*∣ = 93 edges comprised of ∣*E*^↷^∣ = 35 most abundant discontinuous edges, 18 of which canonical and 17 non-canonical (Fig. 4a), JUMPER identified 33 transcripts, 17 of which have an abundance of at least 0.001 as determined by SALMON (Fig. 4b). A subset of eight transcripts are canonical, containing at most one discontinuous edge with the 5ʹ junction in TRS-L and the first ATG downstream of the 3ʹ junction coinciding with the start codon of a known ORF. These canonical transcripts correspond to ORF1ab, ORF3a, E, M, ORF7a, ORF7b, ORF8, N. In particular, ORF1ab (abundance of 0.008) corresponds to the complete viral genome, necessary for viral replication. Notably, ORF10 is the only missing ORF in the identified transcriptome, which is in line with previous studies^3,5^ that did not find evidence for active transcription of ORF10.

As mentioned, JUMPER inferred nine non-canonical transcripts, denoted as X, Xʹ, 1abʹ, Sʹ, 3aʹ, 6ʹ, Eʹ, 7b* and Nʹ. Among these, transcripts 1abʹ, Sʹ, 3aʹ and 6ʹ encode for the 1ab polypeptide, spike protein S, accessory protein 3a and accessory protein 6, respectively. Transcripts X and Xʹ both contain the discontinuous edge going from position 68 to 15774, with the latter containing an additional discontinuous edge from position 26256 to 26284. The 5ʹ end of the common discontinuous edge occurs within TRS-L, whereas the 3ʹ end occurs in the middle of ORF1b but is out of frame with respect to the starting position of ORF1b (13468). Specifically, the start codon ‘ATG’ downstream of the 3ʹ end is located at position 15812 and occurs within nsp12 (RdRp) and the first stop codon is located at position 15896, encoding for a peptide sequence of 28 amino acids. Interestingly, when we examined the reference genome, we observed matching sequences ‘GAACTTTAA’ near the 5ʹ and 3ʹ junctions of the discontinuous edge common to X and Xʹ, possibly explaining why the viral RdRp generated this jump (Supplementary Fig. 9a, b). Strikingly, both matching sequences are conserved within the *Sarbecovirus* subgenus but not in other subgenera of the *Betacoronavirus* genus (Supplementary Fig. 9a, c). To further corroborate this transcript, we examined short- and long-read SARS-CoV-2 sequencing samples from the NCBI Sequence Read Archive (SRA). Specifically, we looked for the presence of reads potentially originating from transcript X focusing on high-quality samples with 100 or more leader-spanning reads (reads whose 5ʹ end maps to the TRS-L region). We say a read *r* supports a transcript *T* if the discontinuous edges of *r* exactly match those of *T*, i.e. *π*(*r*) ⊆ *π*(*T*) and ∣*σ*^⊕^(*r*)∣ = ∣*σ*(*T*)∣ (Supplementary Fig. 10). We found ample support for transcript X in both short- and long-read samples on SRA, with 100 out of 351 short-read samples and 81 out of 653 long-read samples having more than 0.1% of leader-spanning reads supporting transcript X (Supplementary Fig. 11). We note that although this discontinuous transcription event was also observed in ref. ^5^, the authors found no evidence of this transcript leading to a protein product in the ribo-seq data. Further research into a potentially regulatory function of this transcript is required.

As stated, the difference between transcripts X and Xʹ is that the latter includes an additional discontinuous edge, corresponding to a short jump of ~27 nucleotides between positions 26256 and 26284. This is an in-frame deletion inside ORF E, resulting in the loss of nine amino acids that span the N-terminal domain (four amino acids) and the transmembrane domain (five amino acids) of the E protein^37^. A similar in-frame deletion of 24 nucleotides (from position 26259 to 26284) was observed by Finkel et al.^5^ that resulted in the loss of a subset of eight out of the nine amino acids in the deletion that we observed. Furthermore, it is possible that this common deletion is being selected for during passage in Vero E6 cells, which were used by both Kim et al.^3^ and Finkel et al.^5^. Non-canonical transcripts Sʹ, 3aʹ and Eʹ also contain the same discontinuous edge from position 26256 to 26284. While transcript Eʹ produces a version of protein E with nine missing amino acids, transcripts Sʹ and 3aʹ produce complete viral proteins S and 3a, respectively. Non-canonical transcript 6ʹ differs from the canonical transcript 6, containing a jump from position 27886 to 27909. This jump is downstream of ORF6 and therefore does not disrupt the translation of accessory protein 6. Similarly, transcript 1abʹ has a single jump from position 26779 to 26817, which is downstream of the ORF1ab gene and therefore will yield the complete polypeptide 1ab. Transcript 7b*, on the other hand, has a single discontinuous edge from position 71 to 27762. The start codon ‘ATG’ downstream of the 3ʹ end occurs at position 27825, maintaining the frame of 7b, and thus leading to an N-terminal truncation^3^ of 23 amino acids. Interestingly, transcript 7b and transcript 7b* appear with similar abundances in our solution. Finally, transcript Nʹ has one canonical discontinuous edge from TRS-L (position 65) to the transcription regulating body sequence (TRS-B) region corresponding to ORF N (position 28255) and an additional jump from position 28525 to 28577, which leads to an in-frame deletion of 17 amino acids in the N-terminal RNA-binding domain^38,39^ of ORF N. Thus, with the exception of transcripts X and Xʹ, the non-canonical transcripts identified by JUMPER either produce complete viral proteins (1abʹ, Sʹ, 3aʹ, 6ʹ), contain in-frame deletions in the middle of known proteins (Eʹ, Nʹ) or produce N-terminally truncated proteins (7b*).

One of the major findings of the Kim et al. paper^3^ is that the SARS-CoV-2 transcriptome is highly complex owing to numerous non-canonical discontinuous transcription events. Strikingly, our results show that these non-canonical transcription events do not significantly change the resulting proteins. Indeed, we find that four out of the nine non-canonical transcripts produce a complete known viral protein and the total abundance of the predicted transcripts that produce a complete known viral protein is 0.968. Moreover, these predicted transcripts account for more than 90% of the reads in the sample according to the estimates provided by SALMON.

Typically, reads from short-read sequencing samples are not long enough to contain more than one discontinuous edge. As a result, short-read data can only provide direct evidence for transcripts with closely spaced discontinuous edges. For instance, we observed ample support (63485 short reads) for the predicted non-canonical transcript Eʹ, which has two discontinuous edges (69, 26237) and (26256, 26284), in short-read data due to the close proximity of the two discontinuous edges (i.e. the discontinuous edges are only 26256 − 26237 = 19 nucleotides apart). The other non-canonical transcripts with multiple discontinuous edges, i.e. Xʹ, Sʹ, 3aʹ, 6ʹ and Nʹ, have edges that are too far apart to be spanned by a single short read. Using the long-read sequencing data of this sample, we detected supporting long reads that span the exact set of discontinuous edges of all 9 non-canonical transcripts (Fig. 4c). Moreover, we found support for the canonical transcripts as well (Supplementary Fig. 12). Thus, all transcripts identified by JUMPER from the short-read data are supported by direct evidence in the long-read data.

In summary, using JUMPER, we reconstructed a detailed picture of the transcriptome of a short-read sequencing sample of Vero cells infected by SARS-CoV-2. While existing methods failed to recall even the reference transcriptome, JUMPER identified transcripts encoding for all known viral protein products. In addition, our method predicted non-canonical transcripts, whose presence we subsequently validated on a long-read sequencing sample of cells from the same cell line.

### Viral transcript assembly in SARS-CoV-2-infected A549 cells with and without treatment

To demonstrate that JUMPER can be used to understand the effect of drugs on the viral transcriptome, we analyzed a recent dataset by Blanco et al.^25^ who studied the host transcriptional response to SARS-CoV-2 and other viral infections using various cell lines. We focused on A549 lung alveolar cell line samples that were sequenced after 24 h of SARS-CoV-2 infection. There are a total of eight samples, four technical replicates that were pre-treated with ruxolitinib for 1 h before the infection and four technical replicates that were untreated. Ruxolitinib is a JAK1 and 2 kinase inhibitor, which blocks type-I interferon (IFN-I) signaling necessary to engage cellular antiviral defenses^40,41^. Specifically, the four samples without treatment are SRR11573904 (median depth of 86), SRR11573905 (median depth of 85), SRR11573906 (median depth of 89), and SRR11573907 (median depth of 89), and the four samples treated with ruxolitinib are SRR11573924 (median depth of 90), SRR11573925 (median depth of 91), SRR11573926 (median depth of 91), and SRR11573927 (median depth of 92). We used fastp to trim the short reads (trimming parameter set to 10 nucleotides) and we aligned the resulting reads using STAR in two-pass mode. We ran JUMPER with the 35 most abundant discontinuous edges in the segment graph. Similarly to the previous analysis, we restricted our attention to transcripts identified by JUMPER that have more than 0.001 abundance as estimated by SALMON^23^.

**Fig. 5:**
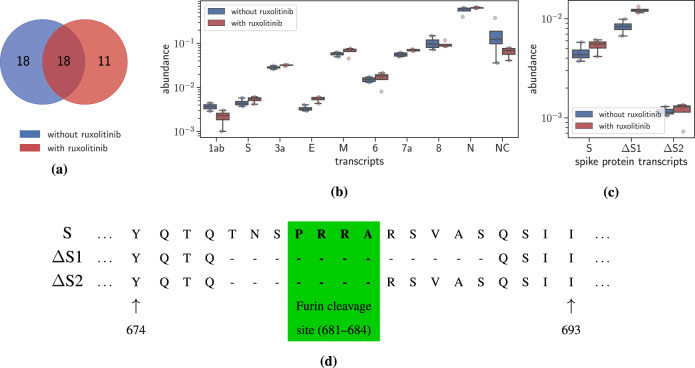
JUMPER enables analysis of drug response in SARS-CoV-2-infected cells^25^ at the transcript level. **a** A Venn diagram showing the number of transcripts reconstructed from four samples with and four samples without treatment with ruxolitinib (i.e. two groups of four technical replicates). Supplementary Fig. 13 shows the distribution of the 18 transcripts that are common between samples with and without treatment while Supplementary Table 4 describes these transcripts. **b** Abundance of the transcripts yielding canonical proteins in the samples along with ‘NC’ depicting the abundance of the non-canonical transcripts. **c** Abundance of the transcripts yielding the spike protein (S) and its variants ΔS1 and ΔS2 whose structure is described in (**d**). Box plots show the median and the interquartile range (IQR), and the whiskers denote the lowest and highest values within 1.5 times the IQR from the first and third quartiles, respectively.

SCALLOP, run with default parameters (Supplementary Note C.2), identified at most two transcripts for each sample encoding for different variants of ORF N. JUMPER identified a total of 47 transcripts across the eight samples, with 18 of these transcripts present in both ruxolitinib treated and untreated samples (Supplementary Fig. 13a, c). We observed that samples with pre-treatment of ruxolitinib cumulatively have fewer transcripts compared to the number of transcripts from samples without any treatment (29 vs. 36 transcripts, Fig. 5a). Strikingly, all the transcripts that are present in two or more samples were also present across the two groups of samples (treated and untreated). Focusing on the 18 common transcripts, Supplementary Fig. 11d shows the total number of samples that contain each of these 18 transcripts. A subset of 13 out of these 18 transcripts produce all known canonical viral proteins except 7b. Figure 5b shows the abundance of the transcripts yielding functional proteins in the samples along with ‘NC’ depicting the abundance of transcripts producing either non-canonical or non-functional viral proteins. The abundance of the canonical transcripts, except 1ab, is slightly higher in samples with treatment compared to the samples without treatment. Consequentially, the abundance of non-canonical transcripts is lower in samples with treatment compared to samples without treatment.

There are five non-canonical transcripts, including ∇M, NC1, and NC2, which do not encode for known SARS-CoV-2 proteins but are explained by matching motifs near the 5ʹ and 3ʹ ends of the non-canonical discontinuous edges, described in Supplementary Table 4, potentially mediating the jump made by the RdRp to generate these transcripts. Specifically, while transcript ∇M contains a canonical discontinuous edge from the leader to the known TRS-B region of M, it also contains an out-of-frame deletion such that the transcript yields a 116 amino acids long protein which matches the M protein for the first 87 amino acids (total length of protein M is 222 amino acids). Both transcripts NC1 and NC2 contain only one jump with the 5ʹ end within ORF1a. The 3ʹ end of the jump lies within ORF7b and ORF N for transcript NC1 and transcript NC2, respectively. The remaining two non-canonical transcripts, ΔS1 and ΔS2, have in-frame deletions in the region that encodes for the spike protein.

ΔS1 contains an in-frame jump from position 23593 to 23630 resulting in a 12 amino-acid in-frame deletion, while ΔS2 contains a jump from position 23593 to 23615, which results in a 7 amino-acid in-frame deletion in the spike protein (Fig. 5d). Both these deletions overlap with the furin cleavage site (FCS), highlighted in Fig. 5d, which has been the focus of several recent studies^4,42,43^. The authors of ref. ^4^ deduced that the deletion of the FCS enhances the ability of the virus to enter Vero cells and is selected for during passage in Vero E6 cells, a cell line that lacks a working type-I interferon response. The observation of ΔS1 and ΔS2 in infected A549 cell samples can be explained by the fact that Blanco et al.^25^ propagated SARS-CoV-2 in Vero E6 cells prior to the infection of the A549 cells. Figure 5c shows that pre-treatment with ruxolitinib leads to an increase in the abundance of the three transcripts, S (median increase from 0.004 to 0.005), ΔS1 and ΔS2 (median increase from 0.0011 to 0.0012), with the increase being most significant for ΔS1 (median increase from 0.008 to 0.012) with a *p* value of 0.015 with the Mann−Whitney *U* test. This shows that the response of different transcripts of the virus to treatment of drugs can differ significantly. In summary, we find that JUMPER enables transcript-level analysis of the viral response to drug treatments.

### Viral transcript assembly in SARS-CoV-1- and MERS-CoV-infected cells

To show the generalizability of our method, we considered two other coronaviruses, SARS-CoV-1 and MERS-CoV. We describe the results for two SARS-CoV-1-infected cell samples here and the analysis of three MERS-CoV-infected cell samples is described in Supplementary Note C.5.

**Fig. 6:**
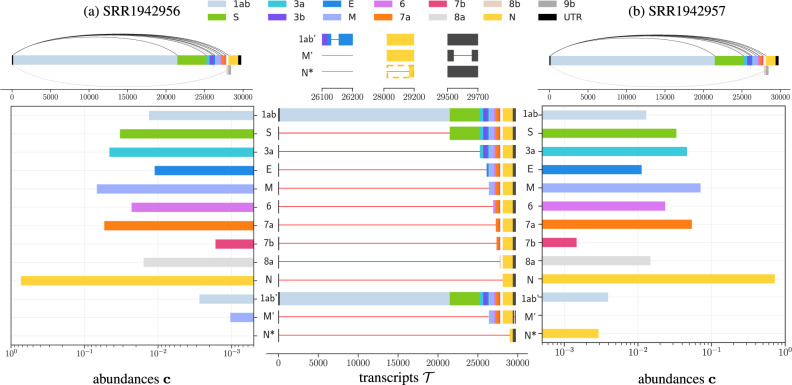
JUMPER identifies canonical and non-canonical transcripts that recur in two short-read sequencing samples of SARS-CoV-1-infected Calu-3 cells^24^. For both the samples, **a** SRR1942956 and **b** SRR1942957, we show the segment graph, with canonical (above) and non-canonical (below) discontinuous edges. We also show the predicted transcripts and their abundances in the two samples with a zoomed-in view of the non-canonical transcripts 1ab', M' and N*. UTR: untranslated region.

We analyzed two published samples of human Calu-3 cells infected with SARS-CoV-1^24^, SRR1942956 and SRR1942957, with a median depth of 21,358 and 20,991, respectively. These two samples originate from the same SRA project (‘PRJNA279442’) whose metadata states that both samples were sequenced 24 h after infection. We used fastp to trim the short reads (trimming parameter set to 10 nucleotides) and we aligned the resulting reads using STAR in two-pass mode. We ran JUMPER with the 35 most abundant discontinuous edges in the segment graph. As observed previously, SCALLOP only identified a single transcript corresponding to ORF N in both the samples. By contrast, JUMPER reconstructed 25 transcripts in sample SRR1942956 and 26 transcripts for sample SRR1942957. Similarly to the previous analysis, we discuss the transcripts identified by JUMPER that have more than 0.001 abundance as estimated by SALMON. There are 13 such transcripts for sample SRR1942956 and 13 such transcripts for sample SRR1942957 (Fig. 6).

SARS-CoV-1 has a genome of length 29,751 bp, and consists of 13 ORFs (1ab, S, 3a, 3b, E, M, 6, 7a, 7b, 8a, 8b, N and 9b), two more than SARS-CoV-2. For both samples, JUMPER identified canonical transcripts corresponding to all the ORFs of SARS-CoV-1 except ORF3b, ORF8b and ORF9b (Fig. 6). Notably, ORF8b and ORF9b share transcription regulating body sequences (TRS-B) with ORF8a and ORF N respectively^44^. More specifically, ORF9b (from position 28130 to 28426) is nested within ORF N (from position 28120 to 29388) with start codons only 10 nucleotides apart and consequently shares the same TRS-B as ORF N. ORF8b (from position 27864 to 28118) intersects with ORF8a (from 27779 to 27898) and previous studies have failed to validate a TRS-B region for ORF8b^44^. One possible way that these ORFs are translated is due to ribosome leaky scanning, which was also hypothesized to lead to ORF7b translation in SARS-CoV-2 ^5^. This explains why JUMPER was unable to identify transcripts that directly encode for 8b and 9b. Regarding ORF3b, JUMPER did identify a canonical transcript corresponding to 3b in both samples, but the SALMON estimated abundances (0.00044 for SRR1942956 and 0.0005 for SRR1942957) for these transcripts were below the cut-off value of 0.01. Finally, we note that the relative abundances of the canonical transcripts are consistent for the two samples (Fig. 6) and ranked in the same order (Supplementary Fig. 14), with ORF7b being the least abundant and ORF N having the largest abundance, in line with the observations in SARS-CoV-2-infected cells described in the previous sections.

Figure 6 shows the three non-canonical transcripts predicted by JUMPER in the two SARS-CoV-1 samples, designated as 1abʹ, Mʹ and N*. Since these non-canonical transcripts are in very low abundance, we see some discrepancy in the prediction between the two samples. The first non-canonical transcript 1abʹ with a single short discontinuous edge from position 26131 to 26156 is detected in both samples and has a very low abundance compared to the canonical transcript 1ab (0.0133 for 1ab vs. 0.002 for 1abʹ in SRR1942956, and 0.013 for 1ab vs. 0.0039 for 1abʹ in SRR1942956). Since the discontinuous edge occurs downstream of the stop codon of 1ab (position 21492), the 1abʹ transcript encodes for the complete polypeptide 1ab. The second non-canonical transcript Mʹ has two discontinuous edges: a canonical discontinuous edge from TRS-L (position 65) to TRS-B of ORF M (position 26351) and a non-canonical discontinuous edge from 29542 to 29661 in the 3ʹ untranslated region (UTR). As such, this transcript encodes for the complete M protein. This transcript is detected in SRR1942956 with a very low abundance of 0.001 and is detected at an even lower abundance of 0.0008 in SRR1942957, which is below the cut-off threshold of 0.001. The third non-canonical transcript, denoted by N*, has a single discontinuous edge from position 65 to 29003. While JUMPER and SALMON detected this transcript only in sample SRR1942957 with a low abundance of 0.003, we do observe 119 reads in SRR1942956 (compared to 151 reads in SRR1942957) that support this edge, suggesting that N* might be present in the latter sample at too small of an abundance to be detected. Transcript N* is interesting because the first ‘ATG’ downstream of the 3ʹ end of its discontinuous edge occurs at position 29071 maintaining the frame of N (which starts at position 28120). Thus transcript N* encodes for an N-terminally truncated version of protein N with 105 amino acids (while protein N is composed of 422 amino acids) and only contains part of the C-terminal dimerization domain^38^ of protein N. This is similar to transcript 7b* in the SARS-CoV-2-infected Vero cell sample, which yields a N-terminal truncated version of protein 7b. Detection of non-canonical transcripts such as Eʹ and 7b* in SARS-CoV-2 and Nʹ in SARS-CoV-1 suggests that generation of N-terminally truncated proteins might be a common feature in coronaviruses.

In summary, JUMPER can used to to reconstruct the transcriptome of all viruses in *Nidovirales* and lead to discovery of novel viral transcripts and corresponding viral proteins. While this section focused on SARS-CoV-1, we observed similar results for MERS-CoV samples, where JUMPER reconstructed transcripts corresponding to all the ORFs with well-supported TRS-B sites along with consistent abundances across the three samples (see Supplementary Note C.5).

## Discussion

In this paper, we formulated the DISCONTINUOUS TRANSCRIPT ASSEMBLY (DTA) problem of reconstructing viral transcripts from short-read RNA-seq data of coronaviruses. The discontinuous transcription process exhibited by the viral RNA-dependent RNA polymerase (RdRp) is distinct from alternative splicing observed in eukaryotes. Our proposed method, JUMPER, is specifically designed to reconstruct the viral transcripts generated by discontinuous transcription and is therefore able to outperform existing transcript assembly methods such as SCALLOP and STRINGTIE, as we have shown in both simulated and real data.

For real-data analysis, we used publicly available short-read and long-read sequencing data of the same sample of SARS-CoV-2-infected Vero cells^3^. We performed transcript assembly using the short-read sequencing data and used the long-read data for validation. JUMPER was able to identify transcripts encoding for all known viral proteins except ORF10, which has been shown to have little support of active transcription in previous studies^3,5^. Moreover, we predicted nine non-canonical transcripts that are well supported by long-read sequencing data.

Furthermore, we demonstrated that JUMPER enables transcript-level quantitative analysis of viral response to treatment with drugs. More specifically, we analyzed eight samples of A549 lung alveolar cells infected by SARS-CoV-2, four of which were pre-treated with ruxolitinib for 1 h before infection^25^. JUMPER identified one variant of the spike protein, with a 12 amino acid deletion overlapping with the furin cleavage site, that showed statistically significant increase in expression in samples that were pre-treated with ruxolitinib. We also showed the versatility of JUMPER by considering two additional coronaviruses, SARS-CoV-1 and MERS-CoV. For two samples of Calu-3 cells infected by SARS-CoV-1 and three samples of Calu-3 cells infected by MERS-CoV^24^, JUMPER reconstructed all the canonical transcripts with distinct TRS-B regions and additionally predicted the presence of non-canonical transcripts encoding for either complete or truncated versions of known viral proteins.

There are several avenues for future work. First, JUMPER currently is only applicable to data obtained using technologies that limit positional bias such as oligo(dT) amplification, which targets the poly(A) tail at the 3ʹ end of messenger RNAs. We plan to extend our current model to account for positional and sequencing biases in the data. Doing so will enable us to assemble transcriptomes from sequencing samples that used SARS-CoV-2-specific primers, which form the majority of currently available data. Second, we currently make the assumption of a fixed read length that is much smaller than the length of viral transcripts. We will relax this assumption in order to support long-read sequencing data that have variable read lengths, similar to previous methods such as Bayesembler^16^ and Scallop-LR^45^. Third, we plan to study the effect of mutations (including single-nucleotide variants as well as indels) on the transcriptome. Along the same lines, there is evidence of within-host diversity in COVID-19 patients^46–51^. It will be interesting to study whether this diversity translates to distinct sets of transcripts and abundances within the same host. Fourth, there are possibly multiple optimal solutions to the DTA problem that present equally likely viral transcripts with different relative abundances in the sample. A useful direction of future work is to explore the space of optimal solutions similar to the work done in ref. ^28^. Finally, the approach presented in this paper can extended to the general transcript assembly problem. Although JUMPER can be used for transcript assembly of individual eukaryotic genes (see Supplementary Note C.4), it does not currently support assembly across multiple genes. The extension of the current approach can be facilitated by using the topological ordering of the nodes in a general splice graph that does not have a unique Hamiltonian path, unlike the segment graph considered in the DTA problem. We envision this will facilitate efficient use of combinatorial optimization tools such as integer linear programming to transcript assembly problems.

## Methods

### Combinatorial characterization of solutions

Equation (1) defines the probability $\Pr ({{{{{{{\mathcal{R}}}}}}}}| {{{{{{{\mathcal{T}}}}}}}},{{{{{{{\bf{c}}}}}}}})$ in terms of the observed reads *r* and their induced paths *π*(*r*) ⊆ *E*(*G*) in the segment graph *G*. The authors in ref. ^28^ use this characterization of reads as paths in a general splice graph to account for ambiguity in the transcript of origin for the reads. For a general splice graph, such a characterization is required to capture all the possible observed reads. However, in our setting, where the segment graph *G* is a DAG with a unique Hamiltonian path, it is possible to describe each read and each transcript uniquely in a more concise form. Each path in the segment graph is characterized by a set of non-overlapping discontinuous edges. To describe this, we introduce the following definition.

#### Definition 3

Two edges (**v** = [*v*^−^, *v*^+^], **w** = [*w*^−^, *w*^+^]) and (**x** = [*x*^−^, *x*^+^], **y** = [*y*^−^, *y*^+^]) of *Goverlap* if the open intervals (*v*^+^, *w*^−^) and (*x*^+^, *y*^−^) intersect, i.e. $({v}^{+},{w}^{-})\cap ({x}^{+},{y}^{-})\,\ne\, {{\emptyset}}$.

For any transcript *T* corresponding to an **s** − **t** path in *G*, for which we are only given its discontinuous edges *σ*(*T*), the continuous edges of *T* are uniquely determined by *G* and *σ*(*T*). That is, the continuous edges of *T* equal precisely the subset of continuous edges *E*^→^ that do not overlap with any of the discontinuous edges in *σ*(*T*). Conversely, given an **s** − **t** path *π*(*T*) of *G* the corresponding set of discontinuous edges is given by *σ*(*T*) = *π*(*T*) ∩ *E*^↷^. Thus, we have the following proposition with the proof in Supplementary Note B.1.

#### Proposition 1

*There is a bijection between subsets of discontinuous edges that are pairwise non-overlapping and* **s** − **t** *paths in* *G*.

In a similar vein, rather than characterizing a read *r* by its induced path *π*(*r*) ⊆ *E* in the segment graph, we characterize a read *r* by a pair (*σ*^⊕^(*r*), *σ*^⊖^(*r*)) of characteristic discontinuous edges. Here, *σ*^⊕^(*r*) is the set of discontinuous edges that must be present in any transcript that could generate read *r*, i.e. *σ*^⊕^(*r*) = *π*(*r*) ∩ *E*^↷^. Conversely, *σ*^⊖^(*r*) is the set of discontinuous edges that must be absent in any transcript that could generate read *r* due to the unidirectional nature of RdRp transcription. Thus, the set *σ*^⊖^(*r*) consists of discontinuous edges *E*^↷^⧹*σ*^⊕^ that overlap with an edge in *π*(*r*). Clearly, while ${\sigma }^{\oplus }(r)\cap {\sigma }^{\ominus }(r)={{\emptyset}}$, it need not hold that *σ*^⊕^(*r*) ∪ *σ*^⊖^(*r*) equals *E*^↷^ (see Fig. 2b). Formally, we define (*σ*^⊕^(*r*), *σ*^⊖^(*r*)) as follows.

#### Definition 4

The *characteristic discontinuous edges* of a read *r* are a pair (*σ*^⊕^(*r*), *σ*^⊖^(*r*)) where *σ*^⊕^(*r*) is the set of discontinuous edges present in read *r*, i.e. *σ*^⊕^(*r*) = *π*(*r*) ∩ *E*^↷^, and ${\sigma }_{i}^{\ominus }$ is the set of discontinuous edges (**v** = [*v*^−^, *v*^+^], **w** = [*w*^−^, *w*^+^]) ∈ *E*^↷^⧹*σ*^⊕^(*r*) that *overlaps* with an edge (**x** = [*x*^−^, *x*^+^], **y** = [*y*^−^, *y*^+^]) in *π*(*r*).

We have the following result with the proof given in Supplementary Note B.1.

#### Proposition 2

*Let* *G* *be a segment graph,* *T* *b e a transcript and* *r* *be a read. Then,* *π*(*T*) ⊇ *π*(*r*) *if and only if* $\sigma (T)\supseteq {\sigma }^{\oplus }(r)\,{{{and}}}\,\sigma (T)\cap {\sigma }^{\ominus }(r)={{\emptyset}}$.

Hence, we may rewrite the likelihood $\Pr ({{{{{{{\mathcal{R}}}}}}}}| {{{{{{{\mathcal{T}}}}}}}},{{{{{{{\bf{c}}}}}}}})$ as

2 $$\mathop{\prod }\limits_{j=1}^{n}\frac{1}{\mathop{\sum }\nolimits_{b = 1}^{k}{c}_{b}{L}_{b}}\mathop{\sum}\limits_{i\in X({{{{{{{\mathcal{T}}}}}}}},{\sigma }_{j}^{\oplus },{\sigma }_{j}^{\ominus })}{c}_{i}.$$

where ${{{{{{{\mathcal{R}}}}}}}}=\{{r}_{1},\ldots ,{r}_{n}\}$, ${{{{{{{\mathcal{T}}}}}}}}=\{{T}_{1},\ldots ,{T}_{k}\}$, **c** = [*c*_1_, …, *c*_*k*_], and where $X({{{{{{{\mathcal{T}}}}}}}},{\sigma }_{j}^{\oplus },{\sigma }_{j}^{\ominus })$ be the subset of indices *i* corresponding to transcripts ${T}_{i}\in {{{{{{{\mathcal{T}}}}}}}}$ where $\sigma ({T}_{i})\supseteq {\sigma }_{j}^{\oplus }$ and $\sigma ({T}_{i})\cap {\sigma }_{j}^{\ominus }={{\emptyset}}$. Note that the only difference between Eq. (2) and the formulation in Eq. (1) is the way that the candidate transcripts of origin for a given read are described. In Eq. (1), they are described as paths in the segment graph whereas in Eq. (2), they are described by sets of pairwise non-overlapping discontinuous edges in the segment graph. This leads to the following theorem.

#### Theorem 1

*For any alignment* ${{{{{{{\mathcal{R}}}}}}}}$*, transcripts* ${{{{{{{\mathcal{T}}}}}}}}$ *and abundances* **c**, *Eqs.* (1) *and* (2) *are identical.*

Although we have described the formulation for single-end reads, this characterization is applicable to paired-end and even synthetic long reads. Moreover, our implementation provides support for both single-end and paired-end read samples with a fixed read length. The above characterization using discontinuous edges allows us to reduce the number of terms in the likelihood function since multiple reads can be characterized by the same characteristic discontinuous edges. We describe this in detail in the next section.

### JUMPER: a progressive heuristic for the DTA problem

To solve the DTA problem, we use the results of the previous section to write a more concise form of the likelihood. Specifically, let ${{{{{{{\mathcal{S}}}}}}}}=\{({\sigma }_{1}^{\oplus },{\sigma }_{1}^{\ominus }),\ldots ,({\sigma }_{m}^{\oplus },{\sigma }_{m}^{\ominus })\}$ be the set of pairs of characteristic discontinuous edges generated by the reads in alignment ${{{{{{{\mathcal{R}}}}}}}}$. Let **d** = {*d*_1_, ⋯ , *d*_*m*_}, where *d*_*i*_ is the number of reads that map to pair $({\sigma }_{i}^{\oplus },{\sigma }_{i}^{\ominus })\in {{{{{{{\mathcal{S}}}}}}}}$. Using that reads *r* with identical characteristic discontinuous edges (*σ*^⊕^(*r*), *σ*^⊖^(*r*)) have identical probabilities $\Pr (r| {{{{{{{\mathcal{T}}}}}}}},{{{{{{{\bf{c}}}}}}}})$, we obtain the following mathematical program for the log-likelihood ${{{{{{\mathrm{log}}}}}}}\,\Pr ({{{{{{{\mathcal{R}}}}}}}}| {{{{{{{\mathcal{T}}}}}}}},{{{{{{{\bf{c}}}}}}}})$ (see Supplementary Note A.2 for derivation).

3 $$\mathop{\max }\limits_{{{{{{{{\mathcal{T}}}}}}}},{{{{{{{\bf{c}}}}}}}}}\mathop{\sum }\limits_{j=1}^{m}{d}_{j}{{{{{{\mathrm{log}}}}}}}\,\mathop{\sum}\limits_{i\in X({{{{{{{\mathcal{T}}}}}}}},{\sigma }_{j}^{\oplus },{\sigma }_{j}^{\ominus })}{c}_{i}-n{{{{{{\mathrm{log}}}}}}}\,\mathop{\sum }\limits_{b=1}^{k}{c}_{b}{L}_{b}$$

4 $$\begin{array}{ll}\,{{\mbox{s.t.}}}\,&\pi ({T}_{i})\,{{\mbox{is an}}}\,{{{{{{{\bf{s}}}}}}}}-{{{{{{{\bf{t}}}}}}}}\,{{\mbox{path}}}\,\\ &\,{{\mbox{in the segment graph}}}\,G,\forall i\in [k],\end{array}$$

5 $$\mathop{\sum }\limits_{i=1}^{k}{c}_{i}=1,$$

6 $${c}_{i}\ \ge \ 0,\quad \forall i\in [k].$$

Observe that the first sum (over reads) is concave and the second sum (over transcripts) is convex. Since we are maximizing, our objective function would ideally be concave. In Supplementary Note B.1, we prove the following lemma, which enables us to remove the second term using a scaling factor for the relative abundances **c** that does not alter the solution space.

#### Lemma 1

*Let* *D* > 0 *be a constant,* ${\overline{c}}_{i}({{{{{{{\bf{c}}}}}}}})={c}_{i}D/\mathop{\sum }\nolimits_{j = 1}^{k}{c}_{j}{L}_{j}$ *and* ${c}_{i}(\overline{{{{{{{{\bf{c}}}}}}}}})={\overline{c}}_{i}/\mathop{\sum }\nolimits_{j = 1}^{k}{\overline{c}}_{j}$ *for all* *i* ∈ [*k*]*. Then,* $({{{{{{{\mathcal{T}}}}}}}},{{{{{{{\bf{c}}}}}}}}=[{c}_{1}(\overline{{{{{{{{\bf{c}}}}}}}}}),\ldots ,{c}_{k}(\overline{{{{{{{{\bf{c}}}}}}}}})])$ *is an optimal solution for* (3)−(6) *if and only if* $({{{{{{{\mathcal{T}}}}}}}},\overline{{{{{{{{\bf{c}}}}}}}}}=[{\overline{c}}_{1}({{{{{{{\bf{c}}}}}}}}),\ldots ,{\overline{c}}_{k}({{{{{{{\bf{c}}}}}}}})])$ *is an optimal solution for*

7 $$\mathop{\max }\limits_{{{{{{{{\mathcal{T}}}}}}}},\overline{{{{{{{{\bf{c}}}}}}}}}}\ \mathop{\sum }\limits_{j=1}^{m}{d}_{j}{{{{{{\mathrm{log}}}}}}}\,\mathop{\sum}\limits_{i\in X({{{{{{{\mathcal{T}}}}}}}},{\sigma }_{j}^{\oplus },{\sigma }_{j}^{\ominus })}{\overline{c}}_{i}$$

8 $$\begin{array}{ll}\,{{{s.t.}}}\,\ &\pi ({T}_{i})\,{{{is\, an}}}\,{{{{{{{\bf{s}}}}}}}}-{{{{{{{\bf{t}}}}}}}}\,{{{path}}}\,\\ &\,{{{in\, the\, segment\, graph}}}\,G,\forall i\in [k],\end{array}$$

9 $$\mathop{\sum }\limits_{i=1}^{k}{\overline{c}}_{i}{L}_{i}=D,$$

10 $${\overline{c}}_{i}\ \ge \ 0,\quad \forall i\in [k].$$

We formulate the mathematical program given in Lemma 1 as a mixed integer linear program. More specifically, we encode (i) the composition of each transcript *T*_*i*_ as a set *σ*(*T*_*i*_) of non-overlapping discontinuous edges, (ii) the abundance *c*_*i*_ and length *L*_*i*_ of each transcript *T*_*i*_, (iii) the total abundance ${\sum }_{i\in X({{{{{{{\mathcal{T}}}}}}}},{\sigma }_{j}^{\oplus },{\sigma }_{j}^{\ominus })}{c}_{i}$ of transcripts supported by characteristic discontinuous edges $({\sigma }_{j}^{\oplus },{\sigma }_{j}^{\ominus })$, and (iv) a piecewise linear approximation of the ${{{{{{\mathrm{log}}}}}}}\,$ function using a user-specified number *h* of breakpoints. We will describe (i) and (ii) in the following and refer to Supplementary Note B.2 for (iii) and (iv).

#### Transcript composition

We begin modeling (8), which states that each transcript *T*_*i*_ must correspond to an **s** − **t** path in the segment graph *G*. Using Proposition 1, we introduce binary variables ${{{{{{{\bf{x}}}}}}}}\in {\{0,1\}}^{| {E}^{\curvearrowright}| \times k}$ to encode the presence of discontinuous edges in each of the *k* **s** − **t** paths corresponding to the *k* transcripts in ${{{{{{{\mathcal{T}}}}}}}}$. For any discontinuous edge *e* = (**v** = [*v*^−^, *v*^+^], **w** = [*w*^−^, *w*^+^]), let *I*(*e*) denote the open interval (*v*^+^, *w*^−^) between the two segments **v** and **w**. By Proposition 1, it must hold that $I(e)\cap I(e^{\prime} )={{\emptyset}}$ for any two distinct discontinuous edges *e* and $e^{\prime}$ assigned to the same transcript. To encode this, we impose

$$\begin{array}{ll}&{x}_{e,i}+{x}_{e^{\prime} ,i}\ \le \ 1,\quad \forall i\in [k],e,e^{\prime} \in {E}^{\curvearrowright }\\ &\,{{\mbox{s.t.}}}\,e\,\ne\, e^{\prime} ,I(e)\cap I(e^{\prime} )\,\ne\, {{\emptyset}}.\end{array}$$

#### Transcript abundance and length

We introduce non-negative continuous variables **c** = [*c*_1_, …, *c*_*k*_] that encode the abundance of the *k* transcripts. The scale of these abundances depends on the choice of *D*. We choose *D* = ℓ* where ℓ* is the length of the shortest **s** − **t** path in the segment graph *G*. Substituting *D* = ℓ* into (9) yields $\mathop{\sum }\nolimits_{i = 1}^{k}{c}_{i}{L}_{i}={\ell }^{* }$.

Since ${c}_{i}{L}_{i}\ \le \ \mathop{\sum }\nolimits_{j = 1}^{k}{c}_{j}{L}_{j}={\ell }^{* }$ and *L*_*i*_ ≥ ℓ*, we have that *c*_*i*_ ≤ 1. To model the product *c*_*i*_*L*_*i*_ of the length *L*_*i*_ of a transcript *T*_*i*_ and its abundance *c*_*i*_, we focus on individual discontinuous edges *e*. For any discontinuous edge *e* = (**v** = [*v*^−^, *v*^+^], **w** = [*w*^−^, *w*^+^]), let *L*(*e*) = *w*^−^ − *v*^+^ be the length of the interval. Observe that

$${c}_{i}{L}_{i}={c}_{i}L-{c}_{i}\mathop{\sum}\limits_{e\in \sigma ({T}_{i})}L(e)={c}_{i}L-\mathop{\sum}\limits_{e\in {E}^{\curvearrowright }}{c}_{i}{x}_{e,i}L(e).$$

We introduce continuous variables *z*_*e*_ ∈ [0, 1]^*k*^ and encode the product *z*_*e*,*i*_ = *c*_*i*_*x*_*e*,*i*_ for all *e* ∈ *E*^↷^ as

$$\begin{array}{ll}{z}_{e,i}\ \le \ {c}_{i},\hfill &\forall i\in [k],\hfill\\ {z}_{e,i}\ \le \ {x}_{e,i},\hfill &\forall e\in {E}^{\curvearrowright },i\in [k],\\ {z}_{e,i}\ \ge \ {c}_{i}+{x}_{e,i}-1,\quad &\forall e\in {E}^{\curvearrowright },i\in [k].\end{array}$$

Therefore, we may represent $\mathop{\sum }\nolimits_{i = 1}^{k}{c}_{i}{L}_{i}={\ell }^{* }$ as

11 $$\mathop{\sum }\limits_{i=1}^{k}{c}_{i}L-\mathop{\sum }\limits_{i=1}^{k}\mathop{\sum}\limits_{e\in {E}^{\curvearrowright }}{z}_{e,i}L(e)={\ell }^{* }.$$

The resulting formulation has *O*(∣*E*^↷^∣*k* + ∣*E*^↷^∣*m* + *mh*) variables, where *h* is the user-specified number of breakpoints used in the piecewise linear approximation of the ${{{{{{\mathrm{log}}}}}}}\,$ function. This number includes ∣*E*^↷^∣*k* binary variables. The number of constraints is *O*(*k*∣*E*∣^2^ + ∣*E*∣*km*).

#### Progressive heuristic

In practice, the number of discontinuous edges in the segment graph is inflated due to ambiguity in the exact location at which the RdRp jumps as well as sequencing and alignment errors. This leads to large number of binary variables in our MILP (we have *k* ⋅ ∣*E*^↷^∣ binary variables) which can make the MILP intractable. In order to approximately solve the problem with large values of *k*, we implement a progressive heuristic. Our heuristic takes as input the alignment ${{{{{{{\mathcal{R}}}}}}}}$ and an integer *k*, which is the maximum number of transcripts in the solution. At each iteration *p* ≤ *k*, we are given a set ${{{{{{{\mathcal{T}}}}}}}}$ of *p* − 1 previously computed transcripts and seek a new transcript $T^{\prime}$ by solving the MILP (see Supplementary Note B.3 for details) using function SOLVEILP with additional constraints to fix the values of the variables that encode the presence/absence of discontinuous edges for the transcripts in ${{{{{{{\mathcal{T}}}}}}}}$. The resulting reduction in number of binary variables from ∣*E*^↷^∣*k* to ∣*E*^↷^∣ improves the running time of the MILP. As an additional optimization, we re-estimate the abundances of a new set ${{{{{{{\mathcal{T}}}}}}}}^{\prime}$ of transcripts. This set contains all transcripts in ${{{{{{{\mathcal{T}}}}}}}}$ as well as additional transcripts corresponding to all possible subsets of discontinuous edges $\sigma (T^{\prime} )$ of the newly identified transcript $T^{\prime}$, identified by the function EXPAND. We solve a linear program (see Supplementary Note B.3 for details) with function SOLVELP to re-estimate the abundances ${{{{{{{\bf{c}}}}}}}}^{\prime}$ of ${{{{{{{\mathcal{T}}}}}}}}^{\prime}$, retaining only the top *p* transcripts *T*_*i*_ from ${{{{{{{\mathcal{T}}}}}}}}^{\prime}$ with the largest abundances *c*_*i*_*L*_*i*_. We terminate upon convergence, i.e. if ${{{{{{{\mathcal{T}}}}}}}}={{{{{{{\mathcal{T}}}}}}}}^{\prime}$, or if the number *p* of iterations reaches the number *k*. We note that a segment graph *G* with ∣*E*^↷^∣ discontinuous edges induces a space of ${2}^{| {E}^{\curvearrowright }| }$, thus providing a theoretical upper bound for *k*. However, in practice, we typically restrict our attention to the set of transcripts that exceed a minimum abundance threshold, resulting in a much smaller value for *k*. Algorithm 1 provides the pseudo code of the progressive heuristic implemented in JUMPER. The details of the subproblems SOLVEILP and SOLVELP are given in Supplementary Note B.3.

##### Algorithm 1

JUMPER($${{{{{{{\mathcal{R}}}}}}}}$$, *k*)

**1** $$({{{{{{{\mathcal{T}}}}}}}},{{{{{{{\bf{c}}}}}}}})\leftarrow ({{\emptyset}},[])$$

**2** **for** *p* ← 1 **to** *k* **do**

**3**        $$T^{\prime} \leftarrow$$ SOLVEILP $$({{{{{{{\mathcal{T}}}}}}}})$$

**4**        $${{{{{{{\mathcal{T}}}}}}}}^{\prime} \leftarrow {{{{{{{\mathcal{T}}}}}}}}\cup$$ EXPAND $$(T^{\prime} )$$

**5**        $${{{{{{{\bf{c}}}}}}}}^{\prime} \leftarrow$$ SOLVELP $$({{{{{{{\mathcal{T}}}}}}}}^{\prime} )$$

**6**        Sort $({{{{{{{\mathcal{T}}}}}}}}^{\prime} ,{{{{{{{\bf{c}}}}}}}}^{\prime} )$ s.t. ${L}_{i}{c}_{i}^{\prime}\ \ge \ {L}_{i+1}{c}_{i+1}^{\prime}$ for all $i\in \{1,\ldots ,| {{{{{{{\mathcal{T}}}}}}}}^{\prime} | -1\}$

**7**        $$({{{{{{{\mathcal{T}}}}}}}}^{\prime} ,{{{{{{{\bf{c}}}}}}}}^{\prime} )\leftarrow (\{{T}_{1},\ldots ,{T}_{p}\},[{c}_{1}^{\prime},\ldots ,{c}_{p}^{\prime}])$$

**8**        **if** $${{{{{{{\mathcal{T}}}}}}}}^{\prime} \ne {{{{{{{\mathcal{T}}}}}}}}$$ **then**

**9**                 $$({{{{{{{\mathcal{T}}}}}}}},{{{{{{{\bf{c}}}}}}}})\leftarrow ({{{{{{{\mathcal{T}}}}}}}}^{\prime} ,{{{{{{{\bf{c}}}}}}}}^{\prime} )$$

**10**        **else**

**11**                 **return** $$({{{{{{{\mathcal{T}}}}}}}},{{{{{{{\bf{c}}}}}}}})$$

**12** **return** $$({{{{{{{\mathcal{T}}}}}}}},{{{{{{{\bf{c}}}}}}}})$$

#### Implementation details

Matching core sequences that mediate the discontinuous transcription by RdRp lead to ambiguity in precise location of breakpoint during alignment of spliced reads. Therefore, in practice we observe multiple discontinuous edges with closely spaced 5ʹ and 3ʹ breakpoints. Moreover, false-positive discontinuous edges are introduced due to sequencing and alignment errors. We use a threshold on the number of spliced reads supporting a discontinuous edge to filter false-positive edges with low support. This parameter can also be used to reduce computational burden and focus on the highly expressed transcripts in the sample. A discussion on the choice of the thresholding parameter Λ is provided in Supplementary Note B.4.

### Reporting summary

Further information on research design is available in the Nature Research Reporting Summary linked to this article.

## Supplementary information

Supplementary Information

Reporting Summary

## Data Availability

The sequencing data deposited by Kim et al.^3^ into the Open Science Framework (OSF) at 10.17605/OSF.IO/8F6N9 were analyzed. The accession numbers of data available on SRA analyzed in this study are—SRR11573904, SRR11573905, SRR11573906, SRR11573907, SRR11573924, SRR11573925, SRR11573926, SRR11573927, SRR1942956, SRR1942957, SRR10357372, SRR10357373, and SRR10357374. All the simulated data generated in this study have been deposited to the Illinois Databank and are available at https://databank.illinois.edu/datasets/IDB-6667667. The analyzed and processed real and simulated data and results are available at https://github.com/elkebir-group/Jumper-data.

## Code availability

The code has been deposited on Github at https://github.com/elkebir-group/Jumper^52^.
