# Supplementary material for: Jumper enables discontinuous transcript assembly in coronaviruses
Source: Nat Commun. 2021 Nov 18;12:6728. doi: 10.1038/s41467-021-26944-y (PMC8602663; doi:10.1038/s41467-021-26944-y)
Supplement: Supplementary file 1 — Supplementary Information [file 41467_2021_26944_MOESM1_ESM.pdf]

Supplementary Material for “JUMPER Enables Discontinuous Transcript Assembly in Coronaviruses”

Palash Sashittal[1], Chuanyi Zhang[2], Jian Peng[1,3], and Mohammed El-Kebir[1,*]

[1]Dept. of Computer Science, University of Illinois at Urbana-Champaign, IL 61801

[2]Dept. of Electrical & Computer Engineering, University of Illinois at Urbana-Champaign, IL 61801

[3]College of Medicine, University of Illinois at Urbana-Champaign, IL 61801

[*]Correspondence: melkebir@illinois.edu

Contents

A Supplementary Notes **3**

A.1 Relation to previous work 3

A.1.1 Mixed-integer linear programming for transcript assembly 4

A.2 Likelihood model for DISCONTINUOUS TRANSCRIPT ASSEMBLY 6

A.3 Relevance of transcript assembly on short-read sequencing data 8

B Supplementary Methods **9**

B.1 Recharacterization of solutions using discontinuous edges 9

B.2 Mixed integer linear program 13

B.3 JUMPER: progressive heuristic for the DTA problem 16

B.4 Filtering false positive discontinuous edges 18

B.5 Extension to paired-end and synthetic long reads 19

C Supplementary Results **19**

C.1 Simulation pipeline 19

C.2 SCALLOP arguments 20

C.3 STRINGTIE arguments 21

C.4 Human gene simulations 21

C.5 Transcript assembly of MERS-CoV samples 23
C.6 Supplementary results figures 25

A Supplementary Notes

A.1 Relation to previous work

Cufflinks [1], one of the first reference-based transcript assembly methods, uses an overlap graph, in which nodes represent read fragments and two nodes are connected if the corresponding fragments overlapped and if every implied intron in one fragment was matched with an identical intron in the other fragment. The authors find the smallest set of transcripts that together support all reads by reducing the transcript assembly problem to finding maximum matching in a weighted bipartite graph. The abundances of the transcripts are then estimated using a statistical model in which the probability of observing a read fragment is a linear function of the abundance of the transcript from which it originated.

The next generation of reference-based transcript assembly methods employ a splice graph rather than an overlap graph. Specifically, they aim to decompose the splice graph constructed for the given alignment into a parsimonious set of transcripts, each corresponding to a distinct source-to-sink path in the splice graph. Bayesembler [2] is a sampling-based method that performs Bayesian inference for transcript assembly and quantification. Specifically, it uses Gibbs sampling method to sample the posterior distribution of transcripts and their abundances for a given set of read fragments. The final transcriptome is selected based on the frequency at which transcripts are observed during the sampling process. Candidate transcripts are generated by exhaustive enumeration of all source-sink paths in the splice graph and parsimony in the solution is promoted using a Bernoulli prior on the expression of transcripts. CLASS [3] also enumerates all source-sink paths and scores them using a linear programming formulation. Subsequently, a subset of transcripts is selected parsimoniously explains the observed reads. IsoLASSO [4], on the other hand, promotes parsimony by performing L1 regularization on the expression level of the transcripts while minimizing the discrepancy between the predicted and observed read coverage. Note that the above methods enumerate all source-to-sink paths as part of their inference. The total number of such paths can become prohibitively large for splice graphs with many edges. JUMPER does not enumerate all source-to-sink paths but rather uses a compact formulation that efficiently represents the space of all discontinuous transcripts.

Several transcript assembly methods, similarly to JUMPER, employ a greedy iterative approach to reconstruct the transcriptome. STRINGTIE [5] performs greedy decomposition of the splice graph into paths, by iteratively removing the heaviest path and updating the edge weights of the remaining splice graph using a max-flow formulation. SCALLOP [6] generates a parsimonious set of transcripts while minimizes the deviation in read coverage and using paired-end reads as *phasing paths* in the underlying splice graph. The splice graph is iteratively decomposed into source-sink paths such that each phasing path appears as a whole in

at least one of the reconstructed transcripts. Our method, JUMPER, also progressively builds the transcriptome from an underlying segment graph, a constrained version of the splice graph. However, we maximize a likelihood-based objective function that describes the probability of observing single-end or paired-end reads from the reconstructed transcripts.

We use a mixed integer linear program (MILP) to solve the transcript assembly problem. In the following section, we compare JUMPER to existing MILP methods for transcript assembly and describe our main advancements over these methods.

A.1.1 Mixed-integer linear programming for transcript assembly

Mixed-integer linear programming has been used in both reference-based transcript assembly [7, 8] and de novo transcript assembly [9–11] methods. Here we compare our method to two reference-based transcript assembly methods that utilize mixed-integer linear programming, MITIE [7] and MULTITRANS [8].

First, we describe the differences in the way transcripts are represented in the three methods – JUMPER, MITIE and MULTITRANS. Each of these methods describe transcripts as paths in the underlying splice or segment graph, but are encoded differently in the MILP. MULTITRANS enumerates all source-sink paths in the underlying splice graph and introduces a one binary variable to encode the presence of each path. As such, in the worst case, the number of binary variables scale exponentially with the number of edges in the splice graph. MITIE, on the other hand, encodes each transcript as a binary vector $[u_i]$ of size $|E|$, where E is the edge set of the splice graph and $u_i = 1$ if and only if i-th edge in the splice graph is part of the transcript. Constraints are introduced ensure that binary vectors for each transcript correspond to paths on the splice graph. Briefly, for each edge in the graph, a constraint is introduced that enforces that if that edge is part of the transcript then any of the preceding edges are also part of the transcript. JUMPER uses a similar encoding with binary variables, except it only uses vector of size $|E^\frown| < |E|$ where $E^\frown$ is the set of discontinuous edges in the segment graph. Moreover, constraints are only introduced for each pair of overlapping discontinuous edges. This leads to fewer binary variables and constraints than the encoding used by MITIE.

Table 1 shows the number of $\mathbf{s}$-$\mathbf{t}$ paths in the simulated instances is an order of magnitude higher compared to the number of edges in the segment graph of simulated instances generated in this study. As such, we do not expect MULTITRANS to scale for large segment graphs with many edges since it introduces a binary variable for each $\mathbf{s}$-$\mathbf{t}$ path in the graph. Also, the number of discontinuous edges is less than half of the total number of edges which shows that JUMPER introduces significantly fewer binary variables compared to MITIE, in the MILP. The contrast is even more drastic on the real short-read sequencing data of

seed	rep	$\lvert E\rvert$	$\lvert E^{\rightarrow}\rvert$	$\lvert E^{\frown}\rvert$	number of $\mathbf{s}$-$\mathbf{t}$ paths
0	1	43	25	18	192
0	2	43	25	18	192
0	3	43	25	18	192
0	4	43	25	18	192
0	5	43	25	18	192
1	1	39	23	16	216
1	2	39	23	16	216
1	3	39	23	16	216
1	4	39	23	16	216
1	5	39	23	16	216
2	1	48	29	19	456
2	2	54	33	21	696
2	3	48	29	19	456
2	4	51	31	20	552
2	5	48	29	19	456
3	1	36	21	15	112
3	2	36	21	15	112
3	3	36	21	15	112
3	4	39	23	16	128
3	5	39	23	16	120
4	1	42	25	17	324
4	2	42	25	17	324
4	3	42	25	17	324
4	4	42	25	17	324
4	5	42	25	17	324

Table 1: Number of continuous edges, discontinuous edges and $\mathbf{s}$-$\mathbf{t}$ paths in the segment graph of simulated instances.

SARS-CoV-2 infected Vero cells [12] we considered in this study. While the segment graph for this sample has 129 edges, out of which only 52 are discontinuous edges, the number of $\mathbf{s}$-$\mathbf{t}$ paths is $1,340,016$.

Second, we describe key contributions of JUMPER compared to the existing MILP methods, MITIE [7] and MULTITRANS [8].

1. In both MITIE and MULTITRANS, the length of the transcripts are not considered in the objective function. In MULTITRANS, the authors do not use a likelihood model and instead minimize the number of segments where the observed and expected coverage differs more than ϵ, where ϵ is a user-defined hyperparameter. In MITIE, the authors use a likelihood model with the assumption that the number of reads generated from a transcript is independent of its length. While this simplification can be valid for instances where different gene isoforms are of roughly the same length, they are not valid for coronaviruses, where the length of discontinuous transcripts vary significantly (length of the longest canonical transcript for SARS-CoV-2 is 29903 bp while the shortest canonical transcript only contains roughly 1710 bp). To that end, in JUMPER, we account for the varying lengths of the transcripts.

2. JUMPER uses the paired-end reads in a consistent probabilistic framework to reconstruct the underlying transcripts and their abundances. MITIE and MULTITRANS, on the other hand, include either penalty terms in the objective function or constraints on the final transcriptome. Specifically, MITIE, introduces a penalty term with additional user-defined hyperparameters in the objective function such that solutions in which combinations of segments supported by paired-end reads are part of the predicted transcripts. MULTITRANS introduces a constraint that each paired-end read is supported by at least one transcript in the solution.

A.2 Likelihood model for DISCONTINUOUS TRANSCRIPT ASSEMBLY

We use the segment graph G to compute the probability $\Pr(\mathcal{R} \mid \mathcal{T}, \mathbf{c})$ of observing the alignment $\mathcal{R}$ given transcripts $\mathcal{T}$ and abundances $\mathbf{c}$. We follow the generative model described in [13], which has been extensively used for transcription quantification [14–16]. Let the set $\mathcal{R}$ of reads be $\{1, \ldots, r_n\}$ and the set $\mathcal{T}$ of transcripts be $\mathcal{T} = \{T_1, \ldots, T_k\}$ with lengths $L_1, \ldots, L_k$ and abundances $\mathbf{c} = [c_1, \ldots, c_k]$. In line with current literature, reads $\mathcal{R}$ are generated independently from transcripts $\mathcal{T}$ with abundances $\mathbf{c}$. Further, we must marginalize over the set of transcripts $\mathcal{T}$ as the transcript of origin of any given read is typically unknown, due to $\ell \ll L$. Thus,

$$\Pr(\mathcal{R} \mid \mathcal{T}, \mathbf{c}) = \prod_{j=1}^{n} \Pr(r_j \mid \mathcal{T}, \mathbf{c})$$

$$= \prod_{j=1}^{n} \sum_{i=1}^{k} \Pr(r_j, Z_{i,j} \mid \mathcal{T}, \mathbf{c})$$

$$= \prod_{j=1}^{n} \sum_{i=1}^{k} \Pr(r_j \mid Z_{i,j}) \Pr(Z_{i,j} \mid \mathcal{T}, \mathbf{c}),$$

where $Z_{i,j}$ is the indicator random variable for the event that T_i is the transcript of origin for read r_j. We denote by $\Pr(r_j \mid Z_{i,j})$ the probability of observing read r_j given that it is generated from transcript T_i and $\Pr(Z_{i,j} \mid \mathcal{T}, \mathbf{c})$ denotes the probability of generating a read from transcript T_i given transcripts $\mathcal{T}$ and abundances $\mathbf{c}$.

Assuming no amplification and sequencing bias, the probability $\Pr(Z_{i,j} \mid \mathcal{T}, \mathbf{c})$ of generating a read from a transcript T_i of length L_i is given by

$$\Pr(Z_{i,j} \mid \mathcal{T}, \mathbf{c}) = \frac{c_i L'_i}{\sum_{j=1}^{k} c_j L'_j},$$

where $L'_i = L_i - \ell$ for $i \in [k]$ is the *effective length* of the transcript T_i. We now derive the probability $\Pr(r_j \mid Z_{i,j})$ of transcript T_i generating read r_j of fixed length ℓ. We do so using the segment graph $G = (V, E)$. Recall that a transcript T must correspond to an $\mathbf{s}$ to $\mathbf{t}$ path in G. Let $\pi(T) \subseteq E$ denote the path corresponding to transcript T. Similarly, each read r induces a path $\pi(r) \subseteq E$ in G. Read r can only be generated by transcript T if $\pi(r) \subseteq \pi(T)$. Hence, the probability of transcript T_i generating a given read r_j is given by

$$\Pr(r_j \mid Z_{i,j}) = \mathbb{1}(\pi(r_j) \subseteq \pi(T_i)) \frac{1}{L'_i},$$

where $L'_i = L_i - \ell$ is the *effective length* of the transcript and $\mathbb{1}$ denotes the indicator function. We assume that the transcripts are much longer than the reads and as such $L'_i / L_i \approx 1$. Putting it all together we get

$$\begin{aligned}
\Pr(\mathcal{R} \mid \mathcal{T}, c) &= \prod_{j=1}^{n} \sum_{i=1}^{k} \Pr(r_j \mid Z_{i,j}) \Pr(Z_{i,j} \mid \mathcal{T}, \mathbf{c}) \\
&= \prod_{j=1}^{n} \sum_{i=1}^{k} \frac{\mathbf{1}\{\pi(r_j) \subseteq \pi(T_i)\}}{L'_i} \cdot \frac{c_i L'_i}{\sum_{b=0}^{k} c_b L'_b} \\
&= \prod_{j=1}^{n} \sum_{i:\pi(T_i) \supseteq \pi(r_j)} \frac{1}{L'_i} \cdot \frac{c_i L'_i}{\sum_{b=0}^{k} c_b L'_b} \\
&= \prod_{j=1}^{n} \frac{1}{\sum_{b=1}^{k} c_b L_b (1 - \ell / L_b)} \sum_{i:\pi(T_i) \supseteq \pi(r_j)} c_i \\
&\approx \prod_{j=1}^{n} \frac{1}{\sum_{b=1}^{k} c_b L_b} \sum_{i:\pi(T_i) \supseteq \pi(r_j)} c_i.
\end{aligned}$$

A.3 Relevance of transcript assembly on short-read sequencing data

Recent years have seen significant development in third-generation sequencing technologies, such as Oxford Nanopore Technologies (ONT) and Pacific Biosciences single-molecule real time (PacBio SMRT) sequencing. Long-reads provided by these technologies during RNA-sequencing provides valuable information regarding the composition of the underlying transcripts in the sample. Nevertheless, we believe assembly using short-read sequencing data is still relevant due to the following reasons.

1. Even with long-read sequencing, the reads often do not span the entire transcript. As such, some assembly is often required even with long-read sequencing data using methods such as Scallop-LR [17].

2. While Nanopore sequencing is cost effective it is more error-prone compared to short-read sequencing. Current cost of Pacbio sequencing, on the other hand, is much higher compared to short-read sequencing with the Illumina [18] [1].

3. Both long-read sequencing technologies have high insertion and deletion rates which can lead to incorrect identification of jumps in the discontinuous transcripts [19]. Long-read sequencing is challenging for applications that require high base-level accuracy, such as transcript assembly and defining intron-exon boundaries [20].

4. Nanopore sequencing suffers from 3' bias while PacBio performs preferential sequencing of smaller fragments (transcripts) which leads to inaccurate estimates in relative expression of genes [21]. Iso-seq, RNA-sequencing platform of PacBio, requires size fractionation, which makes estimation of absolute and relative abundance of transcripts difficult. Moreover, the high per-read cost makes well-replicated differential expression study designs prohibitively expensive [21].

5. There is a lot of short-read sequencing data for SARS-CoV-2 and other coronavirus infected cells publicly available on SRA [22]. As of 8/24/2021, SRA contained 758,921 short-read sequencing samples out of a total of 924,785 SARS-CoV-2 sequencing samples. Methods like JUMPER will enable transcript assembly and differential expression analysis of the assembled transcripts using this data.

[1] The reader is referred to this google sheet with more up-to-date costs of different technologies – `https://docs.google.com/spreadsheets/d/1GMMfhyLK0-q8XkIo3YxlWaZA5vVMuhU1kg41g4xLkXc`

B Supplementary Methods

B.1 Recharacterization of solutions using discontinuous edges

We prove the following two main text propositions.

(Main Text) Proposition 1. There is a bijection between subsets of discontinuous edges that are pairwise non-overlapping and $\mathbf{s}-\mathbf{t}$ paths in G.

Proof. Let Π be the set of $\mathbf{s}-\mathbf{t}$ paths in G. We indicate with Σ the family of subsets of discontinuous edges that are pairwise non-overlapping. Note that $\Sigma \subseteq 2^{E^{\curvearrowright}}$.

For an $\mathbf{s}-\mathbf{t}$ path $\pi \in \Pi$, let $f(\pi)$ be the set of discontinuous edges in π, *i.e.* $f(\pi) = \pi \cap E^{\curvearrowright}$. Since π is an $\mathbf{s}-\mathbf{t}$ path of G, we have that for each edge $(\mathbf{v} = [v^-, v^+], \mathbf{w} = [w^-, w^+]) \in \pi$ it holds that $v^+ \leq w^-$. Therefore, $f(\pi)$ is composed of pairwise non-overlapping disconnected edges.

Now, consider a subset $\sigma \in \Sigma$ of discontinuous edges that are pairwise non-overlapping. We obtain the corresponding $\mathbf{s}-\mathbf{t}$ path $f^{-1}(\sigma)$ by first ordering the edges of σ in ascending order. That is, let $\sigma = \{(\mathbf{v}_1 = [v_1^-, v_1^+], \mathbf{w}_1 = [w_1^-, w_1^+]), \ldots, (\mathbf{v}_{|\sigma|} = [v_{|\sigma|}^-, v_{|\sigma|}^+], \mathbf{w}_{|\sigma|} = [w_{|\sigma|}^-, w_{|\sigma|}^+])\}$ such that $w_i^+ \leq v_{i+1}^-$ for all $i \in \{1, \ldots, |\sigma|-1\}$. For every two consecutive discontinuous edges $(\mathbf{v}_i = [v_i^-, v_i^+], \mathbf{w}_i = [w_i^-, w_i^+])$ and $(\mathbf{v}_{i+1} = [v_{i+1}^-, v_{i+1}^+], \mathbf{w}_{i+1} = [w_{i+1}^-, w_{i+1}^+])$, we include the corresponding subpath of continuous edges from $\mathbf{w}_i$ to $\mathbf{v}_{i+1}$ into $f^{-1}(\sigma)$. In addition, we include the subpath of continuous edges from node $\mathbf{s}$ to node $\mathbf{v}_1$ as well as the subpath from node $\mathbf{w}_{|\sigma|}$ to $\mathbf{t}$ into $f^{-1}(\sigma)$. By construction, $f^{-1}(\sigma)$ is an $\mathbf{s}-\mathbf{t}$ path. □

(Main Text) Proposition 2. Let G be a segment graph, T be a transcript and r be a read. Then, $\pi(T) \supseteq \pi(r)$ if and only if $\sigma(T) \supseteq \sigma^{\oplus}(r)$ and $\sigma(T) \cap \sigma^{\ominus}(r) = \emptyset$.

Proof. **(⇒)** By the premise, $\pi(T) \supseteq \pi(r)$. By definition, $\sigma(T) = \pi(T) \cap E^{\curvearrowright}$. By Definition 4 from the main text, $\sigma^{\oplus}(r) = \pi(r) \cap E^{\curvearrowright}$. As $\pi(T) \supseteq \pi(r)$, we have that $\sigma(T) = \pi(T) \cap E^{\curvearrowright} \supseteq \pi(r) \cap E^{\curvearrowright} = \sigma^{\oplus}(r)$. By definition, $\sigma^{\ominus}(r)$ is the subset of discontinuous edges in $E^{\curvearrowright} \setminus \sigma^{\oplus}(r)$ that overlaps with an edge in $\pi(r)$. Since $\pi(T) \supseteq \pi(r)$, every edge included in $\sigma^{\ominus}(r)$ because of an overlap with an edge in $\pi(r)$ must also overlap with the same edge in $\pi(T)$. Since $\pi(T)$ is an $\mathbf{s}-\mathbf{t}$ path, and thus does not contain pairwise overlapping edges, we infer that $\sigma^{\ominus}(r) \cap \sigma(T) = \emptyset$.

(⇐) By the premise, $\sigma(T) \supseteq \sigma^{\oplus}(r)$ and $\sigma(T) \cap \sigma^{\ominus}(r) = \emptyset$. As $\sigma(T) \supseteq \sigma^{\oplus}(r)$, we have that $\pi(T) \cap E^{\curvearrowright} = \sigma(T) \supseteq \sigma^{\oplus}(r) = \pi(r) \cap E^{\curvearrowright}$. Since $\sigma(T) \cap \sigma^{\ominus}(r) = \emptyset$, we have by Definition 4 from the main text, that no discontinuous edge in $\sigma(T)$ overlaps with any edge in $\pi(r)$. Since $\pi(T)$ is an $\mathbf{s}-\mathbf{t}$ path containing the subset $\sigma^{\oplus}(r)$ of discontinuous edges in $\pi(r)$, it holds that $\pi(T) \cap E^{\rightarrow} \supseteq \pi(r) \cap E^{\rightarrow}$. Finally, as $E^{\curvearrowright} \cup E^{\rightarrow} = E$, $\pi(r) \subseteq E$ and $\pi(T) \subseteq E$, we get $\pi(T) \supseteq \pi(r)$. □

Using this proposition, we derive a simpler form of the likelihood given in Equation 2 in the main text. Let $\mathcal{S} = \{(\sigma_1^{\oplus}, \sigma_1^{\ominus}), \ldots, (\sigma_m^{\oplus}, \sigma_m^{\ominus})\}$ be the set of characteristic discontinuous edges generated by the reads in alignment $\mathcal{R}$. Let $\mathbf{d} = \{d_1, \cdots, d_m\}$ be the number of reads that map to each pair in $\mathcal{S}$. Using that distinct reads r_j and $r_{j'}$ with the same characteristic discontinuous edges $(\sigma^{\oplus}(r_j), \sigma^{\ominus}(r_j)) = (\sigma^{\oplus}(r_{j'}), \sigma^{\ominus}(r_{j'}))$ have the same likelihood in terms of Equation 2 in the main text, we have

$$\Pr(\mathcal{R} \mid \mathcal{T}, \mathbf{c}) = \prod_{j=1}^{n} \frac{1}{\sum_{b=1}^{k} c_b L_b} \sum_{i \in X(\mathcal{T}, \sigma_j^{\oplus}, \sigma_j^{\ominus})} c_i = \prod_{j=1}^{m} \left(\frac{1}{\sum_{b=1}^{k} c_b L_b} \sum_{i \in X(\mathcal{T}, \sigma_j^{\oplus}, \sigma_j^{\ominus})} c_i \right)^{d_j}. \tag{12}$$

Now, taking the logarithm yields

$$\begin{aligned}
\log \Pr(\mathcal{R} \mid \mathcal{T}, \mathbf{c}) &= \sum_{j=1}^{m} d_j \left(\log \left(\frac{1}{\sum_{b=1}^{k} c_b L_b} \right) + \log \sum_{i \in X(\mathcal{T}, \sigma_j^{\oplus}, \sigma_j^{\ominus})} c_i \right) \\
&= -\sum_{j=1}^{m} d_j \left(\log \sum_{b=1}^{k} c_b L_b \right) + \sum_{j=1}^{m} \left(d_j \log \sum_{i \in X(\mathcal{T}, \sigma_j^{\oplus}, \sigma_j^{\ominus})} c_i \right) \\
&= \sum_{j=1}^{m} \left(d_j \log \sum_{i \in X(\mathcal{T}, \sigma_j^{\oplus}, \sigma_j^{\ominus})} c_i \right) - n \log \sum_{b=1}^{k} c_b L_b.
\end{aligned} \tag{13}$$

The goal is the remove the second sum in the above equation, as it is convex and we are maximizing. In order to do so, we first prove the following lemma.

Lemma 1. For any given scaling factor $\alpha > 0$, we have that $\log \Pr(\mathcal{R} \mid \mathcal{T}, \mathbf{c}) = \log \Pr(\mathcal{R} \mid \mathcal{T}, \alpha\mathbf{c})$.

Proof.

$$\begin{aligned}
\log \Pr(\mathcal{R} \mid \mathcal{T}, \alpha\mathbf{c}) &= \sum_{j=1}^{m} \left(d_j \log \sum_{i \in X(\mathcal{T}, \sigma_j^{\oplus}, \sigma_j^{\ominus})} \alpha c_i \right) - n \log \sum_{b=1}^{k} \alpha c_b L_b \\
&= \sum_{j=1}^{m} \left(d_j \log \left(\alpha \sum_{i \in X(\mathcal{T}, \sigma_j^{\oplus}, \sigma_j^{\ominus})} c_i \right) \right) - n \log \alpha \sum_{b=1}^{k} c_b L_b \\
&= \sum_{j=1}^{m} d_j \log \alpha + \sum_{j=1}^{m} \left(d_j \log \sum_{i \in X(\mathcal{T}, \sigma_j^{\oplus}, \sigma_j^{\ominus})} c_i \right) - n \log \alpha - n \log \sum_{b=1}^{k} c_b L_b \\
&= n \log \alpha + \sum_{j=1}^{m} \left(d_j \log \sum_{i \in X(\mathcal{T}, \sigma_j^{\oplus}, \sigma_j^{\ominus})} c_i \right) - n \log \alpha - n \log \sum_{b=1}^{k} c_b L_b
\end{aligned}$$

$$= \sum_{j=1}^{m} \left(d_j \log \sum_{i \in X(\mathcal{T}, \sigma_j^{\oplus}, \sigma_j^{\ominus})} c_i \right) - n \log \sum_{b=1}^{k} c_b L_b$$

$$= \log \Pr(\mathcal{R} \mid \mathcal{T}, \mathbf{c}).$$

□

This enables us to prove the following lemma.

(Main Text) Lemma 1. Let $D > 0$ be a constant, $\overline{c}_i(\mathbf{c}) = c_i D / \sum_{j=1}^{k} c_j L_j$ and $c_i(\overline{\mathbf{c}}) = \overline{c}_i / \sum_{j=1}^{k} \overline{c}_j$ for all $i \in [k]$. Then, $(\mathcal{T}, \mathbf{c} = [c_1(\overline{\mathbf{c}}), \ldots, c_k(\overline{\mathbf{c}})])$ is an optimal solution for Eq. (3)-(6) from the main text if and only if $(\mathcal{T}, \overline{\mathbf{c}} = [\overline{c}_1(\mathbf{c}), \ldots, \overline{c}_k(\mathbf{c})])$ is an optimal solution for

$$\max_{\mathcal{T}, \overline{\mathbf{c}}} \sum_{j=1}^{m} d_j \log \sum_{i \in X(\mathcal{T}, \sigma_j^{\oplus}, \sigma_j^{\ominus})} \overline{c}_i \tag{14}$$

$$\text{s.t. } \pi(T_i) \text{ is an } \mathbf{s} - \mathbf{t} \text{ path in the segment graph } G \qquad \forall i \in [k], \tag{15}$$

$$\sum_{i=1}^{k} \overline{c}_i L_i = D, \tag{16}$$

$$\overline{c}_i \geq 0 \qquad \forall i \in [k]. \tag{17}$$

Proof. We will refer to the optimization problem in Eq. (3)-(6) from the main text as P and the optimization problem in Eq. (14)-(17) as Q. Further, we will refer to the objective function in Eq. (3) from the main text as $J(\mathcal{T}, \mathbf{c})$ and the objective function in (14) as $K(\mathcal{T}, \overline{\mathbf{c}})$. Observe that

$$K(\mathcal{T}, \overline{\mathbf{c}}) = \log \Pr(\mathcal{R} \mid \mathcal{T}, \overline{\mathbf{c}}) + n \log \sum_{b=1}^{k} \overline{c}_b L_b$$

$$= J(\mathcal{T}, \overline{\mathbf{c}}) + n \log \sum_{b=1}^{k} \overline{c}_b L_b, \tag{18}$$

where the last equality uses (13).

(⇒) Let $(\mathcal{T}, \mathbf{c})$ be an optimal solution to problem P. We begin by showing that $(\mathcal{T}, \overline{\mathbf{c}})$ is a feasible solution to Q where $\overline{\mathbf{c}} = [\overline{c}_1(\mathbf{c}), \ldots, \overline{c}_k(\mathbf{c})]$. By definition of $\overline{c}_i(\mathbf{c})$, constraints Eq. (16) are satisfied. Hence, $(\mathcal{T}, \overline{\mathbf{c}})$ is a feasible solution to problem Q.

We now show that if $(\mathcal{T}, \mathbf{c})$ is an optimal solution to problem P, then $(\mathcal{T}, \overline{\mathbf{c}})$ is an optimal solution to problem Q. Let $(\mathcal{T}', \overline{\mathbf{c}}')$ be an optimal solution to problem Q. Then, by optimality of $(\mathcal{T}', \overline{\mathbf{c}}')$, we have

$$K(\mathcal{T}', \overline{\mathbf{c}}') \geq K(\mathcal{T}, \overline{\mathbf{c}}). \tag{19}$$

Let $\mathbf{c}' = [c_1(\overline{\mathbf{c}}'), \ldots, c_k(\overline{\mathbf{c}}')]$. Note that $\mathbf{c}'$ satisfies constraints in Eq. (5). Thus $(\mathcal{T}', \mathbf{c}')$ is a feasible solution to problem P. Since $(\mathcal{T}, \mathbf{c})$ is an optimal solution of P, we have

$$J(\mathcal{T}, \mathbf{c}) \geq J(\mathcal{T}', \mathbf{c}'). \tag{20}$$

Since $\mathbf{c}'$ and $\overline{\mathbf{c}}'$ only differ by a positive scaling factor $\alpha = 1/\sum_{i=1}^{k} \overline{c}'_i$, we use Lemma 1 to get $J(\mathcal{T}', \mathbf{c}') = J(\mathcal{T}', \overline{\mathbf{c}}')$. Similar result holds for $\mathbf{c}$ and $\overline{\mathbf{c}}$, *i.e.* $J(\mathcal{T}, \mathbf{c}) = J(\mathcal{T}, \overline{\mathbf{c}})$. Applying this to (20), we get

$$J(\mathcal{T}, \overline{\mathbf{c}}) \geq J(\mathcal{T}', \overline{c}').$$

Using (16) and (18), we get

$$\begin{aligned}
& J(\mathcal{T}, \overline{\mathbf{c}}) && \geq J(\mathcal{T}', \overline{\mathbf{c}}') \\
\Longrightarrow\ & K(\mathcal{T}, \overline{\mathbf{c}}) - n \log \sum_{b=1}^{k} \overline{c}_b L_b && \geq K(\mathcal{T}', \overline{\mathbf{c}}') - n \log \sum_{b=1}^{k} \overline{c}'_b L_b \\
\Longrightarrow\ & K(\mathcal{T}, \overline{\mathbf{c}}) - n \log D && \geq K(\mathcal{T}', \overline{\mathbf{c}}') - n \log D \\
\Longrightarrow\ & K(\mathcal{T}, \overline{\mathbf{c}}) && \geq K(\mathcal{T}', \overline{\mathbf{c}}').
\end{aligned} \tag{21}$$

Finally, using (19) and (21), we get $K(\mathcal{T}, \overline{\mathbf{c}}) = K(\mathcal{T}', \overline{\mathbf{c}}')$. Hence, $(\mathcal{T}, \overline{\mathbf{c}})$ is an optimal solution of Q.

(⇐) Let $(\mathcal{T}, \overline{\mathbf{c}})$ be an optimal solution to problem Q. We begin by showing that $(\mathcal{T}, \mathbf{c})$ is a feasible solution to P where $\mathbf{c} = [c_1(\overline{\mathbf{c}}), \ldots, c_k(\overline{\mathbf{c}})]$. By definition of $c_i(\overline{\mathbf{c}})$, constraints in Eq. (5) of the main text are satisfied. Hence, $(\mathcal{T}, \mathbf{c})$ is a feasible solution to problem P.

Next, we need to show that $(\mathcal{T}, \mathbf{c})$ is an optimal solution to problem P. Let $(\mathcal{T}', \mathbf{c}')$ be an optimal solution to problem P.

Then, from the optimality condition, we get

$$J(\mathcal{T}', \mathbf{c}') \geq J(\mathcal{T}, \mathbf{c}). \tag{22}$$

Let $\overline{\mathbf{c}}' = [\overline{c}_1(\mathbf{c}'), \ldots, \overline{c}_k(\mathbf{c}')]$. Note that $\overline{\mathbf{c}}'$ satisties constraint (16) and thus $(\mathcal{T}', \overline{\mathbf{c}}')$ is a feasible solution to problem Q. Using (18) and the fact that $(\mathcal{T}, \overline{\mathbf{c}})$ is an optimal solution of problem Q we get

$$\begin{aligned}
& K(\mathcal{T}, \overline{\mathbf{c}}) && \geq K(\mathcal{T}', \overline{\mathbf{c}}') \\
\Longrightarrow\ & J(\mathcal{T}, \overline{\mathbf{c}}) + n \log \sum_{b=1}^{k} \overline{c}_b L_b && \geq J(\mathcal{T}', \overline{\mathbf{c}}') + n \log \sum_{b=1}^{k} \overline{c}'_b L_b \\
\Longrightarrow\ & J(\mathcal{T}, \overline{\mathbf{c}}) + n \log D && \geq J(\mathcal{T}', \overline{\mathbf{c}}') + n \log D \\
\Longrightarrow\ & J(\mathcal{T}, \overline{\mathbf{c}}) && \geq J(\mathcal{T}', \overline{\mathbf{c}}').
\end{aligned} \tag{23}$$

Observe that $\mathbf{c}'$ and $\overline{\mathbf{c}}'$ only differ by a positive scaling factor $\alpha = D/\sum_{j=1}^{k} c'_j L_j$. Therefore, using Lemma 1, we have $J(\mathcal{T}', \mathbf{c}') = J(\mathcal{T}', \overline{\mathbf{c}}')$. Similarly, for $\mathbf{c}$ and $\overline{\mathbf{c}}$, we have $J(\mathcal{T}, \mathbf{c}) = J(\mathcal{T}, \overline{\mathbf{c}})$. Using this together with (23), we obtain

$$J(\mathcal{T}, \mathbf{c}) \geq J(\mathcal{T}', \mathbf{c}'). \tag{24}$$

Moreover, (22) and (24) simultaneously imply $J(\mathcal{T}, \mathbf{c}) = J(\mathcal{T}', \mathbf{c}')$. Hence, $(\mathcal{T}, \mathbf{c})$ is an optimal solution to problem P.

□

B.2 Mixed integer linear program

In the following, we introduce variables and constraints to encode the following.

(i) The composition of each transcript T_i as a set $\sigma(T_i)$ of non-overlapping discontinuous edges.

(ii) The abundance c_i and length L_i of each transcript T_i.

(iii) The total abundance $\sum_{i \in X(\mathcal{T}, \sigma_j^{\oplus}, \sigma_j^{\ominus})} c_i$ of transcripts supported by characteristic discontinuous edges $(\sigma_j^{\oplus}, \sigma_j^{\ominus})$.

(iv) A piecewise linear approximation of the $\log$ function.

We describe (iii) and (iv) in the following and refer to the Materials and methods section in the main text for (i) and (ii).

Contribution of transcripts to each pair of characteristic discontinuous edges. The objective function has m terms, one corresponding to each pair $(\sigma_j^{\oplus}, \sigma_j^{\ominus}) \in \mathcal{S}$ of characteristic discontinuous edges (see Eq. (7) in the main text). Specifically, each term j equals $d_j \log \sum_{i \in X(\mathcal{T}, \sigma_j^{\oplus}, \sigma_j^{\ominus})} c_i$ where d_j is a constant, for all $j \in [m]$. We introduce non-negative continuous variables $\mathbf{q} = (q_1, \ldots, q_m)$ such that

$$q_j = \sum_{i \in X(\mathcal{T}, \sigma_j^{\oplus}, \sigma_j^{\ominus})} c_i = \sum_{i=1}^{k} \left(c_i \prod_{e \in \sigma_j^{\oplus}} x_{e,i} \prod_{e' \in \sigma_j^{\ominus}} (1 - x_{e',i}) \right), \tag{25}$$

where the last equality uses the characterization of candidate transcripts of origin for a given read described in Proposition 2 of the main text. We introduce continuous variables $\mathbf{y}_j \in [0,1]^k$ that encode the product $y_{j,i} = c_i \prod_{e \in \sigma_j^{\oplus}} x_{e,i} \prod_{e' \in \sigma_j^{\ominus}} (1 - x_{e',i})$. Intuitively, each variable $y_{j,i}$ encodes the contribution

of a transcript T_i for the given characteristic discontinuous edge sets $(\sigma_j^{\oplus}, \sigma_j^{\ominus})$. We linearize the product $c_i \prod_{e \in \sigma_j^{\oplus}} x_{e,i} \prod_{e' \in \sigma_j^{\ominus}} (1 - x_{e',i})$ as follows.

$$
\begin{aligned}
y_{j,i} &\leq c_i, \quad && \forall i \in [k], j \in [m], \\
y_{j,i} &\leq x_{e,i}, \quad && \forall e \in \sigma_i^{\oplus}, i \in [k], j \in [m], \\
y_{j,i} &\leq 1 - x_{e,i}, \quad && \forall e \in \sigma_i^{\ominus}, i \in [k], j \in [m], \\
y_{j,i} &\geq c_i + \sum_{e \in \sigma_j^{\oplus}} x_{e,i} + \sum_{e \in \sigma_j^{\ominus}} (1 - x_{e,i}) - |\sigma_j^{\oplus}| - |\sigma_j^{\ominus}|, \quad && \forall i \in [k], j \in [m].
\end{aligned}
$$

Hence, we have

$$
q_j = \sum_{i=1}^{k} y_{j,i}. \tag{26}
$$

Objective function. The objective function (Eq. (7) in the main text) can be written in terms of continuous variables $\mathbf{q}$ as

$$
J(\mathbf{q}) = \sum_{j=1}^{m} d_j \log q_j,
$$

where d_j is a constant and $\mathbf{q}$ is as in (26). We use the lambda method to approximate our objective ~~method~~function using a piecewise linear function [23]. Following the method described in [23], we partition the domain $(0, 1]$ with h breakpoints $b_1 \leq b_2 \leq \ldots \leq b_h$. We introduce continuous variables $\boldsymbol{\lambda}_j \in [0, 1]^h$ with the constraints

$$
\begin{aligned}
\sum_{o=1}^{h} \lambda_{j,o} &= 1, \quad \forall j \in [m], \\
\sum_{o=1}^{h} b_o \lambda_{j,o} &= q_j, \quad \forall j \in [m].
\end{aligned}
$$

Note that b_o for $o \in [h]$ are constants. Since each of the m terms in the objective function are individually concave and we are maximizing, the adjacency condition of breakpoints does not need to be enforced. For each $j \in [m]$, the log function is then approximated as

$$
\log(q_j) \approx \sum_{o=1}^{h} \lambda_{j,o} \log(b_o),
$$

where $\log(b_o)$ is a constant for each $o \in [h]$. Therefore the objective function we wish to maximize is

$$
\sum_{j=1}^{m} d_j \sum_{o=1}^{h} \lambda_{j,o} \log(b_o).
$$

Note that since we have a log-likelihood objective function, feasibility of the solution requires that $q_j > 0$ for $j \in [m]$. This means that for each characteristic discontinuous edge sets $(\sigma_j^{\oplus}, \sigma_j^{\ominus})$, there must be at least one candidate transcript of origin T_i with non-zero abundance $c_i > 0$. This leads to the solution containing a large number of transcripts and making the problem intractable while also preventing us from finding parsimonious sets of transcripts that support most but not all of the observed reads in the sample. Finding such parsimonious solutions is often desirable since they provide a reasonable explanation of the observed reads while keeping the problem computationally tractable. In order to allow us to generate solutions that can partially explain the observed reads, we slightly modify our objective function. We introduce a new breakpoint $b_0 = 0$ and associated continuous variables $\lambda_{j,0} \in [0, 1]$ for $j \in [m]$ so that

$$\sum_{o=0}^{h} \lambda_{j,o} = 1, \quad \forall j \in [m],$$
$$\sum_{o=0}^{h} b_o \lambda_{j,o} = q_j, \quad \forall j \in [m].$$

The objective function we maximize is

$$\sum_{j=1}^{m} d_j \left(\lambda_{j,0} \log(\delta) + \sum_{o=1}^{h} \lambda_{j,o} \log(b_o) \right),$$

where $\delta > 0$ is a small constant. Note that instead of evaluating the $\log$ function at b_0, we include $\log(\delta)$ which is well defined since $\delta > 0$. In this study, we choose $\delta = b_1/100 = 1/(2^{h-1} \times 100)$ while h is left as the user's choice with default value of 16.

Moreover, the choice of breakpoints to approximate the objective function (Eq. (7) in the main text) can have a significant impact on the accuracy of the MILP solver. As a result, there has been research in efficient methods for choosing optimal breakpoint locations for concave functions, such as recursive descent algorithms [24]. In this work we take a simpler approach, by choosing breakpoints such that their spacing around a given breakpoint is proportional to the local gradient of the objective function. For the log function, this is equivalent to choosing breakpoints such that $b_i = 2^{i-1}/2^{h-1}$. Note that $b_0 = 1/2^h$ while $b_h = 1$.

Number of variables and constraints. The total number of binary variables $\mathbf{x}$ is $|E^{\frown}|k$. Note that $\mathbf{q}$ are auxiliary (intermediate) variables that are uniquely determined by $\mathbf{c}, \mathbf{y}, \mathbf{z}$ and $\boldsymbol{\lambda}$. Therefore, the total number of required continuous variables (*i.e.* $\mathbf{c}$, $\mathbf{y}$, $\mathbf{z}$ and $\boldsymbol{\lambda}$) is $k + mk + |E^{\frown}|k + mh$. The number of constraints is $O(k|E|^2 + |E|km)$. We provide the full MILP for reference.

$$\max \sum_{j=1}^{m} d_j \sum_{o=1}^{h} \lambda_{j,o} \log(b_o)$$

$$
\begin{aligned}
\text{s.t. } & x_{e,i} + x_{e',i} \leq 1, && \forall i \in [k] \text{ and } e, e' \in E^{\frown}, \\
& && \text{s.t. } I(e) \cap I(e') \neq \emptyset, \\
& y_{j,i} \leq c_i, && \forall i \in [k], j \in [m], \\
& y_{j,i} \leq x_{e,i}, && \forall e \in \sigma_j^{\oplus}, i \in [k], j \in [m], \\
& y_{j,i} \leq 1 - x_{e,i}, && \forall e \in \sigma_j^{\ominus}, i \in [k], j \in [m], \\
& y_{j,i} \geq c_i + \sum_{e \in \sigma_j^{\oplus}} x_{e,i} + \sum_{e \in \sigma_j^{\ominus}} (1 - x_{e,i}) - |\sigma_j^{\oplus}| - |\sigma_j^{\ominus}|, && \forall i \in [k], j \in [m], \\
& z_{e,i} \leq c_i, && \forall i \in [k], \\
& z_{e,i} \leq x_{e,i}, && \forall e \in E^{\frown}, i \in [k], \\
& z_{e,i} \geq c_i + x_{e,i} - 1, && \forall e \in E^{\frown}, i \in [k], \\
& \sum_{i=1}^{k} c_i L - \sum_{i=1}^{k} \sum_{e \in E^{\frown}} z_{e,i} L(e) = \ell^*, \\
& \sum_{o=1}^{h} \lambda_{j,o} = 1, && \forall j \in [m], \\
& \sum_{o=1}^{h} b_o \lambda_{j,o} = \sum_{i=1}^{k} y_{j,i}, && \forall j \in [m], \\
& x_{e,i} \in \{0, 1\}, && \forall i \in [k], e \in E^{\frown}, \\
& c_i \geq 0, && \forall i \in [k], \\
& y_{j,i} \geq 0, && \forall j \in [m], i \in [k], \\
& z_{e,i} \geq 0 && \forall e \in E^{\frown}, i \in [k], \\
& \lambda_{j,o} \geq 0, && \forall j \in [m], o \in [h].
\end{aligned}
$$

B.3 JUMPER: progressive heuristic for the DTA problem

Here we describe the subproblems that are solved at each iteration of the greedy heuristic. For a given set of transcripts $\mathcal{T}$ and characteristic discontinuous edge sets $\mathcal{S}$, consider the optimization problem which we denote by P_1,

$$
\max_{T', \mathbf{c}, c'} \sum_{j=1}^{m} d_j \log \left(\sum_{i \in X(\mathcal{T}, \sigma_j^{\oplus}, \sigma_j^{\ominus})} c_i + \mathbf{1}(\sigma_{T'} \supseteq \sigma_j^{\oplus} \wedge \sigma_{T'} \cap \sigma_j^{\ominus} = \emptyset) c' \right) \tag{27}
$$

$$
\text{s.t. } \pi(T') \text{ is an } \mathbf{s} - \mathbf{t} \text{ path in the segment graph } G \tag{28}
$$

$$\sum_{i=1}^{|\mathcal{T}|} \bar{c}_i L_i + c' L' = D, \tag{29}$$

$$c_i \geq 0 \qquad \forall i \in [|\mathcal{T}|] \tag{30}$$

$$c' \geq 0 \qquad . \tag{31}$$

and the following optimization problem denoted by P_2,

$$\max_{\mathbf{c}} \sum_{j=1}^{m} d_j \log \sum_{i \in X(\mathcal{T}, \sigma_j^{\oplus}, \sigma_j^{\ominus})} \bar{c}_i \tag{32}$$

$$\sum_{i=1}^{|T|} c_i L_i = D, \tag{33}$$

$$c_i \geq 0 \qquad \forall i \in [|\mathcal{T}|]. \tag{34}$$

Solution to P_1. We obtain the solution of P_1 by solving the optimization problem given in Eq. (7)-(10) in the main text with additional constraints to fix the values of the variables that encode the presence/absence of discontinuous edges for the transcripts in $\mathcal{T}$. More specifically, for each transcript $T_i \in \mathcal{T}$, we enforce $x_{e,i} = 1$ for each edge $e \in \sigma(T_i)$ and $x_{e,i} = 0$ otherwise. Note that c_i for $T_i \in \mathcal{T}$ are still variables and are solved for in the optimization problem. By doing so, we only solve for the structure of the transcript T' while solving for the abundance of all transcripts.

Solution to P_2. Similar to the approach taken to solve P_1, we fix the values of the variables that encode the presence/absence of discontinuous edges in the transcripts. This results in all the binary variables in the MILP with fixed values rendering the resulting optimization problem a simpler linear program.

Heuristic algorithm. The Algorithm 1 from the main text is re-written here in form of an itemized list.

1. Initialize $\mathcal{T} = \{\}$, $i = 1$
2. Solve P_1 with $\mathcal{T}$ to get a new transcript T' with abundance c'
3. Generate a new set of transcripts $\mathcal{T} \leftarrow \mathcal{T} \cup \text{EXPAND}(T')$ where $\text{EXPAND}(T') = \{T : \sigma(T) \in 2^{\sigma(T')}\}$.
4. Solve P_2 with $\mathcal{T}$ as input
5. Select i transcripts from $\mathcal{T}$. If $i < k$ go to step (2) else return $(\mathcal{T}, \mathbf{c})$

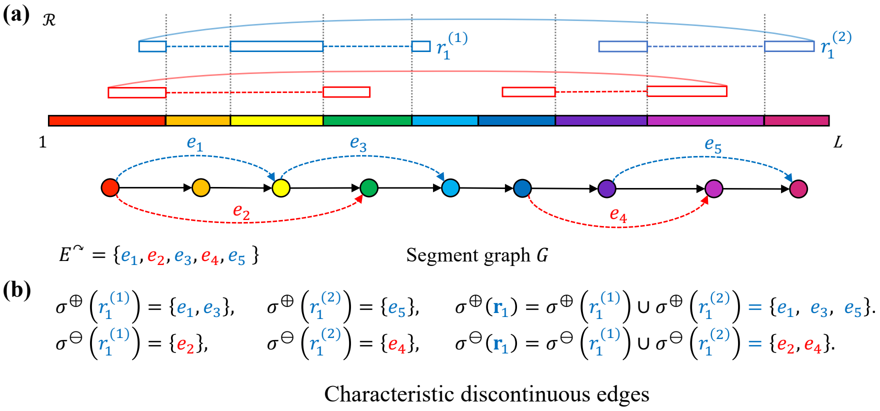

Figure 1: Schematic showing characterization of paired-end reads in JUMPER. (a) Paired-end split reads in an alignment $\mathcal{R}$ with the corresponding segment graph G. (b) The characteristic discontinuous edges $(\sigma^{\oplus}(\mathbf{r}_1), \sigma^{\oplus}(\mathbf{r}_1))$ of paired-end read $\mathbf{r}_1 = \{r_1^{(1)}, r_1^{(2)}\}$ comprises of the characteristic discontinuous edges of the two ends, $r_1^{(1)}$ and $r_1^{(2)}$.

B.4 Filtering false positive discontinuous edges

In practice, we see spurious discontinuous edges in the resulting segment graph due to sequencing and alignment errors. We filter these edges by requiring a minimum number Λ of spliced reads to support each discontinuous edge in the segment graph. The higher the value of Λ, fewer will be the number of edges and nodes in the resulting segment graph.

It is not trivial to infer the optimal value of Λ to remove all false positive discontinuous edges. Several heuristics are used in existing methods to remove spurious splicing events. SCALLOP removes an edge e from its splice graph if the coverage of the exons of either end of the edge is more than $2w(e)^2 + 18$, where $w(e)$ is the number of spliced reads that support the edge e. STRINGTIE on the other hand, terminates its algorithm of assembling transcripts when the coverage of all the paths in the splice graph build from the un-assigned reads drops below a threshold, set by default to 2.5 reads per base-pair. By default, JUMPER requires a support of 100 reads for a discontinuous edge to be included in the segment graph.

Another parameter that can be used to filter false-positive splicing events is the number of discontinuous edges allowed in the segment graph. From tests on simulated instances emulating SARS-CoV-2 samples, we found that focusing on the 35 most abundant discontinuous edges is sufficient to get a summary of the transcriptome and highly expressed canonical and non-canonical transcripts in the sample. A higher value can be used to capture more complexity of the transcriptome. By default, we set this parameter to 35.

B.5 Extension to paired-end and synthetic long reads

In this section we describe the extension of our method to paired-end and synthetic long reads. Recall that each read $r \in \mathcal{R}$ is characterized by a pair $(\sigma^{\oplus}(r), \sigma^{\ominus}(r))$. We can represent a paired-end read $\mathbf{r}$ as a pair $(r^{(1)}, r^{(2)})$ (see Figure 1), where $r^{(1)}$ and $r^{(2)}$ represent the two ends of the paired-end read. In our formulation, this paired-end read will be characterized by the pair

$$\sigma^{\oplus}(\mathbf{r}) = \sigma^{\oplus}(r^{(1)}) \cup \sigma^{\oplus}(r^{(2)}), \quad \sigma^{\ominus}(\mathbf{r}) = \sigma^{\ominus}(r^{(1)}) \cup \sigma^{\ominus}(r^{(2)}).$$

Similarly, a synthetic long read $\mathbf{r}$ with s contiguous component reads can be represented by the tuple $(r^{(1)}, \cdots, r^{(s)})$. Such a synthetic long read $\mathbf{r}$ will be characterized by

$$\sigma^{\oplus}(\mathbf{r}) = \bigcup_{t=1}^{s} \sigma^{\oplus}(r^{(t)}), \quad \sigma^{\ominus}(r) = \bigcup_{t=1}^{s} \sigma^{\ominus}(r^{(t)}).$$

The rest of the formulation is exactly the same as described for single-end reads.

C Supplementary Results

C.1 Simulation pipeline

Our simulations are based on a widely believed model of discontinuous transcription. Briefly, there are two competing models of discontinuous transcription for coronaviruses [25]. Both models agree that the RdRp jump is mediated by matching core-sequences (motifs) present in the TRSs in the viral genome. The only point of difference between the two models is whether discontinuous transcription occurs during the plus-strand synthesis or the minus-strand synthesis. The *negative-sense discontinuous transcription model* [26] proposes that the it is during the minus-strand synthesis that the RdRp performs discontinuous transcription. Transcription is initiated at the 3' end of the plus-strand RNA and the RdRp jumps to the TRS-L region when it reaches a TRS-B region adjacent to a gene, thereby generating a minus-strand subgenomic RNA. The minus-strand subgenomic RNA is then replicated by the RdRp to produce a plus-strand RNA which can be translated to a viral protein. Currently, this model is largely believed to be true due to the considerable experimental support from genetic studies detecting minus-strand subgenomic RNAs [27–31].

We now describe the procedure to simulate transcripts and their abundances following the negative-sense model of discontinuous transcription for a given segment graph. The model is parameterized by the function $p : E \to [0, 1]$. According to the *negative-sense discontinuous transcription* model, the transcription process is modeled as an $\mathbf{t} - \mathbf{s}$ walk in the reverse graph $\bar{G}$ where the direction of each original edge is reversed. At each node the RdRp randomly chooses an outgoing edge to traverse in the reverse graph $\bar{G}$ (which would

be an incoming edge to the node in the original graph G) where the probabilities are given by the function p. Hence, the corresponding constraint on p under the negative-sense discontinuous transcription model is $\sum_{e \in \delta^-(\mathbf{v})} p(e) = 1$. The probabilities are drawn from a Dirichlet distribution with concentration parameter α set to 10 for edges that are present in the path corresponding to any of the canonical transcripts and 1 otherwise. This is done to ensure that canonical transcripts are generated with high enough abundance, making the simulations similar to real data.

The next step of our simulation pipeline is to generate transcripts $\mathcal{T}$ and their abundances $\mathbf{c}$ for the given segment graph. We simulate the transcription process by generating 100,000 $\mathbf{s} - \mathbf{t}$ paths on the segment graph and report the number of unique paths/transcripts $\mathcal{T}$ and their abundances $\mathbf{c}$. We repeat this process to generate 5 independent sets of transcripts and abundances for the positive and the negative model each. Figure 3b in the main text shows the number of transcripts generated from each simulation using the negative-sense discontinuous transcription model. To contrast, the total number of $\mathbf{s} - \mathbf{t}$ paths in the underlying segment graph is 3440.

Once the transcripts are generated, the next step in our pipeline is to simulate the generation and sequencing of RNA-seq data. We use `polyester` [32] for this step as it allows the user to provide the number of reads generated from each transcript. For a given total number n of reads, the number of reads generated from transcript T_i is given by $nc_iL_i / \sum_{j=1}^{k} c_jL_j$ where L_i is the length of the transcript T_i. We use the default parameters for read length ($\ell = 100$) and fragment length distribution (Gaussian with mean $\mu_r = 250$ and standard deviation $\sigma_r = 25$) to generate 3,000,000 reads. For each set of transcript and abundances generated in the previous step of the pipeline, we simulate 5 replicates of the sequencing experiment.

The final step of the simulation pipeline is to align the generated reads to the reference genome NC_045512.2 using `STAR` [33]. The resulting BAM file serves as the input for the transcription assembly methods. To summarize, we generated 5 independent pairs $(\mathcal{T}, \mathbf{c})$ of transcripts and abundances under the negative-sense discontinuous transcription model. For each pair $(\mathcal{T}, \mathbf{c})$ we run 5 simulated sequencing experiments using `polyester` [32]. Therefore, we generated a total of $5 \times 5 = 25$ simulated instances.

C.2 SCALLOP arguments

We use SCALLOP v0.10.4 and the following arguments.

```
scallop -i ${input_bam} -o ${output_assembled}
```

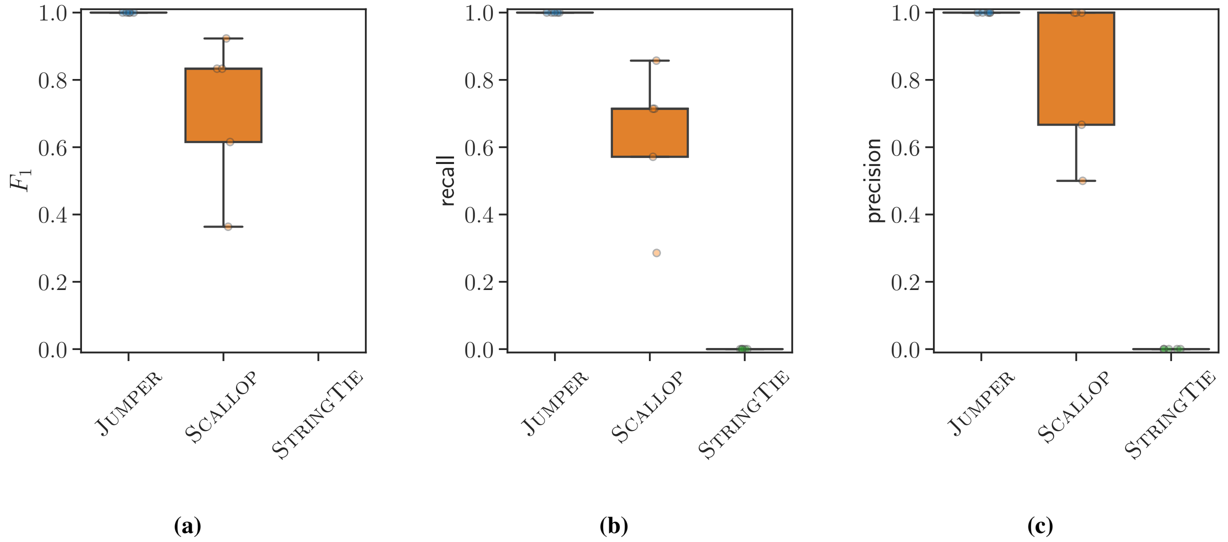

Figure 2: JUMPER outperforms SCALLOP and STRINGTIE for all simulation instances of the FAS gene (on human chromosome 10) with all 7 isoforms of the gene in terms of F_1 score, recall and precision while maintaining a modest running time. (a) F_1 score (b) recall and (c) precision of the three methods for the simulated instances. The ground truth contained seven isoforms of the FAS gene with uniform relative abundances.

C.3 STRINGTIE arguments

We run STRINGTIE v2.1.4 in de novo transcript assembly mode. That is, we do not provide a GFF file to guide assembly. We use the following arguments.

```
stringtie  -o ${output_assembled} -A ${output_abundance} ${input_bam}
```

We noted that STRINGTIE produces incomplete transcripts, *i.e.* all the assembled transcripts did not map to the 5' and 3' end of the reference genome. In our simulations, STRINGTIE was not penalized for this as our evaluation metrics considered only discontinuous edges.

C.4 Human gene simulations

We evaluate the performance of JUMPER, SCALLOP and STRINGTIE on simulated samples of the human gene FAS as well. This gene is located on the long arm of chromosome 10 in humans and encodes the Fas cell surface receptor which leads to programmed cell death if it binds its ligand (Fas ligand). The FAS gene has 15 exons, yielding the following seven isoforms via alternative splicing (`https://www.uniprot.org/uniprot/P25445`).

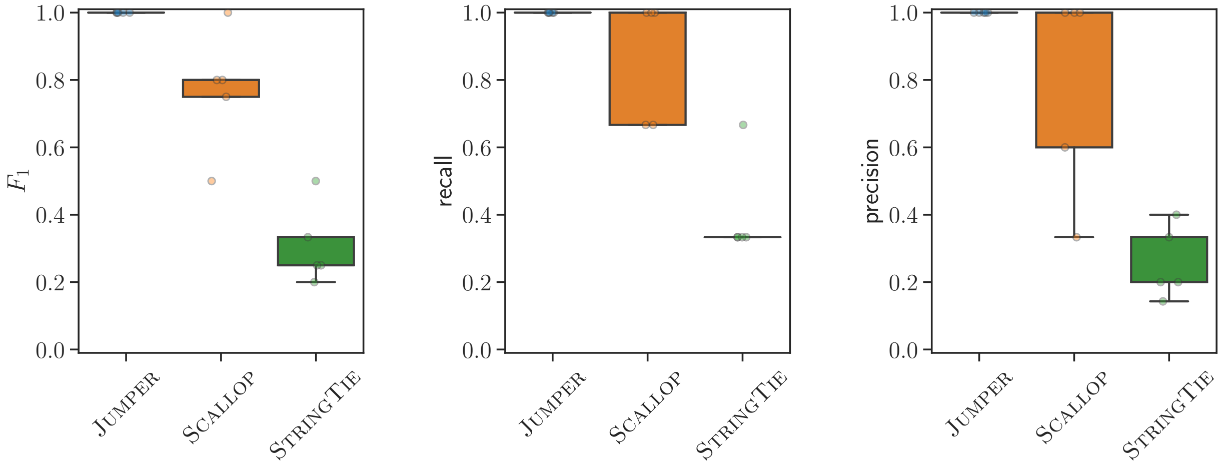

(a) **(b)** **(c)**

Figure 3: JUMPER outperforms SCALLOP and STRINGTIE for all simulation instances of the FAS gene (on human chromosome 10) with only 3 isoforms (P25445-1, P25445-6 and P25445-7) in terms of F_1 score, recall and precision while maintaining a modest running time. (a) F_1 score (b) recall and (c) precision of the three methods for the simulated instances. The ground truth contained three isoforms of the FAS gene with uniform relative abundances.

1. P25445-1 with length of 335aa
   `https://useast.ensembl.org/Homo_sapiens/Transcript/Summary?db=core;g=ENSG00000026103;r=10:88990731-89014619;t=ENST00000652046`
2. P25445-2 with length of 103aa
   `https://uswest.ensembl.org/Homo_sapiens/Transcript/Summary?db=core;g=ENSG00000026103;r=10:88990731-89014619;t=ENST00000484444`
3. P25445-3 with length of 86aa
   `https://uswest.ensembl.org/Homo_sapiens/Transcript/Summary?db=core;g=ENSG00000026103;r=10:88990731-89014619;t=ENST00000479522`
4. P25445-4 with length of 149aa
   `https://uswest.ensembl.org/Homo_sapiens/Transcript/Summary?db=core;g=ENSG00000026103;r=10:88990731-89014619;t=ENST00000494410`
5. P25445-5 with length of 132aa
   `https://uswest.ensembl.org/Homo_sapiens/Transcript/Summary?db=core;`

`g=ENSG00000026103;r=10:88990731-89014619;t=ENST00000492756`

6. P25445-6 with length of 314aa
   `https://uswest.ensembl.org/Homo_sapiens/Transcript/Summary?db=core;g=ENSG00000026103;r=10:88990731-89014619;t=ENST00000357339`
7. P25445-7 with length of 220aa
   `https://uswest.ensembl.org/Homo_sapiens/Transcript/Summary?db=core;g=ENSG00000026103;r=10:88990731-89014619;t=ENST00000355279`

The region between the first and the last exon span position 5001 to 30255 of the FAS gene. We used this region as the reference genome in our simulations[2]. We include the seven isoforms with equal proportion of 1/7 in the ground truth. We add a poly-A tail of length 85 at the end of the reference genome as well as each of the isoforms to emulate the transcription process. We use polyester [32] to simulate the sequencing of 35,000,000 paired-end reads of the sample with a Gaussian fragment length distribution with mean 250 and standard deviation of 25. We simulate 5 replicates of the sequencing experiment. The simulated reads are aligned to the selected region of the FAS gene using `STAR` [33]. The resulting BAM file serves as the input for the transcription assembly methods We evaluate the recall and precision of the three methods focusing on transcripts with abundance of more than 0.01. Figure 2 shows that JUMPER (median F1 score of 1) outperforms SCALLOP (median F1 score of 0.83) in terms of both recall and precision, while STRINGTIE is not able to recall any of the 7 transcripts in the ground truth. We run the simulations again with only 3 of the isoforms, P25445-1, P25445-6 and P25445-7. Figure 3 shows that STRINGTIE is able to perform better with a median recall of 0.33, but still not as well as either SCALLOP (median recall of 1) or JUMPER (median recall of 1).

C.5 Transcript assembly of MERS-CoV samples

MERS-CoV has a genome of length 30119 bp, and consists of 10 ORFs (1ab, S, 3, E, M, 4a, 4b, 5, 8b, N). We ran JUMPER on three published MERS-CoV samples [34], SRR10357372, SRR10357373 and SRR10357374, with a median coverage of 41,999, 36,663 and 45,235 respectively. These samples correspond to MERS-CoV infected Calu-3 cell lines [34]. Similar to previous analyses in this paper, we used `fastp` to trim the short reads (trimming parameter set to 10 nucleotides) and we aligned the resulting reads using `STAR` in two-pass mode. SCALLOP identified at most two canonical transcripts in each of

[2]NCBI reference sequence NG_009089.2: `https://www.ncbi.nlm.nih.gov/nuccore/NG_009089.2?from=5001&to=30255&report=fasta`

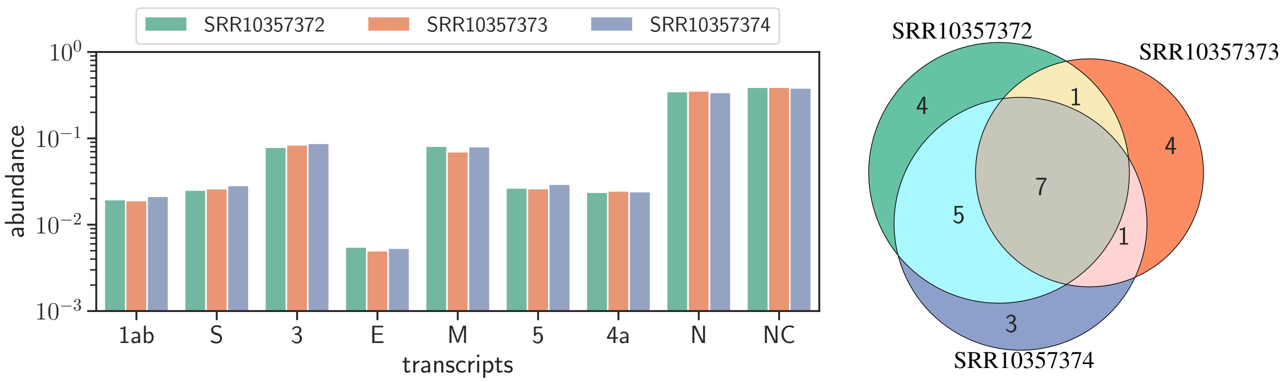

(a) transcript abundances

(b) non-canonical transcripts

Figure 4: JUMPER finds all canonical transcripts and some non-canonical transcripts from three MERS-CoV samples. (a) Abundance of the detected transcripts in the three samples, SRR10357372, SRR10357373 and SRR10357374. (b) A Venn diagram of the non-canonical transcripts reconstructed for each sample showing that there are 7 non-canonical transcripts that are present in all the three samples. Table 2 shows the abundance of the 8 canonical transcripts that are present in all the samples and 14 non-canonical transcripts that are present in more than 1 sample.

the three samples (transcripts corresponding to ORF3 and ORF M in SRR10357372, ORF5 and ORF3 in SRR10357373, and ORF N in SRR10357374). We ran JUMPER with the 35 most abundant discontinuous edges in the segment graph and restrict our attention to transcripts identified by JUMPER that have more than 0.001 abundance as estimated by SALMON [15].

JUMPER reconstructs transcripts corresponding to all canonical ORFs of MERS-CoV in all the samples, except for ORF4b and ORF8b which are the only canonical ORFs that are not preceded by well supported TRS-B regions [35]. The most abundant transcript corresponds to ORF N (median abundance of 0.348), in line with the observations for SARS-CoV-2, while the least abundant canonical transcript encodes for protein E (median abundance of 0.0053). Figure 4a shows, for each sample, the relative abundances of each canonical transcript as well as the total abundances of all non-canonical transcripts. Firstly, we observe that the abundance of each canonical transcript is consistent across the three samples. Secondly, we see that all the three samples have high total abundance of non-canonical transcripts (median total abundance of 0.3908). Figure 4b shows a Venn diagram for the non-canonical transcripts present in the three samples. We see out of the 25 distinct non-canonical transcripts, 7 are present in all the three samples and 14 are present in at least two of the samples. Table 2 shows the abundance of the 8 canonical transcripts present in all the samples and the 14 non-canonical transcripts present in at least two samples. We will now describe the most abundant non-canonical transcripts in each sample.

The most abundant non-canonical transcript in samples SRR10357372 and SRR10357373 is 'NC8', which has a single discontinuous edge from position 1317 (5' end) to 29600 (3' end). The abundance of this

transcript is 0.1019 in sample SRR10357372 and 0.1639 in sample SRR10357372, which is higher than all the canonical transcripts in both the samples except the transcript corresponding to ORF N. The 5' end of the discontinuous edge is in ORF1ab (nsp2 region) and the 3' end is in ORF N. Interestingly the most abundant non-canonical transcript in the third sample SRR10357374 is 'NC12', which has a single discontinuous edge with the same 3' end of 29600 while the 5' end is at position 1297 (also in the nsp2 region of ORF1ab). This transcript has abundance of 0.1486 in sample SRR10357374, higher than all the canonical transcripts in SRR10357374 except the transcript corresponding to ORF N, and 0.0483 in sample SRR10357372. We were not able to attribute the occurrence of transcripts NC8 and NC12 to matching motifs at the 5' and 3' ends of the discontinuous edges. Given the high abundance of these non-canonical transcripts in the sample, further investigation is required to ascertain their function, or whether

C.6 Supplementary results figures

We have the following supplementary figures.

- Figure 5 shows that JUMPER outperforms SCALLOP and STRINGTIE for all simulation instances in terms of F_1 score, recall and precision while maintaining a modest running time.
- Figure 6 shows that JUMPER outperforms SCALLOP and STRINGTIE for varying values of thresholding parameter Λ.
- Figure 7 shows that JUMPER produces better recall and precision when compared to SCALLOP and STRINGTIE for every simulation instance $(\mathcal{T}, \mathbf{c})$.
- Figure 8 shows that SALMON slightly outperform JUMPER in estimating the abundance of transcripts on simulated instances. To compute the correlation of the abundances estimated by each method with the ground truth, we restrict ourselves to the correctly recalled transcripts by JUMPER and renormalize the ground truth abundances accordingly.
- Figure 9 shows that the core sequence observed in the reference genome potentially explaining a non-canonical discontinuous transcription event, and the core sequence corresponding to transcript X is conserved across *Sarbecovirus* species.
- Figure 10 shows an example of a supporting read for a transcript with two discontinuous edges.
- Figure 11 shows that transcript X is supported in both long-read and short-read samples deposited in SRA.

Transcript	Discontinuous Edges	SRR10357372	SRR10357373	SRR10357374	
1ab	-	0.0195	0.0190	0.0213	canonical
S	(59, 21402)	0.0251	0.0261	0.0284	
3	(59, 25518)	0.0789	0.0840	0.0876	
E	(61, 27582)	0.0055	0.0049	0.0053	
M	(58, 27834)	0.0812	0.0699	0.08	
5	(55, 26826)	0.0266	0.0261	0.0294	
4a	(59, 25840)	0.0237	0.0246	0.0241	
N	(53, 28536)	**0.3483**	**0.3542**	**0.34**	
NC1	(62, 28626)	0.0017	0.0016	0.0015	non-canonical
NC2	(65, 29106)	0.0043	0.0029	0.0026	
NC3	(61, 29503)	0.0016	0.0014	0.0015	
NC4	(61, 29582)	0.003	0.0027	0.0029	
NC5	(1727, 28983)	0.016	0.0169	0.0198	
NC6	(2343, 29204)	0.0736	0.1047	0.0575	
NC7	(7120, 24104)	0.0086	0.0088	0.0087	
NC8	(1317, 29600)	**0.1019**	**0.1639**	-	
NC9	(2333, 29203)	0.055	-	0.049	
NC10	(63, 680)<br>(1727, 28983)	0.0019	-	0.0017	
NC11	(59, 21402)<br>(24103, 27938)	0.0011	-	0.0011	
NC12	(1297, 29600)	0.0483	-	**0.1486**	
NC13	(64, 29105)	0.0011	-	0.001	
NC14	(2333, 29150)	-	0.0613	0.0363	

Table 2: Abundance of 8 canonical transcript present in all three MERS-CoV samples and 14 non-canonical transcript present in more than 1 sample. The canonical and non-canonical transcripts with the highest abundance in each sample are highlighted. Figure 4b shows the Venn diagram of all the transcripts in the solution.

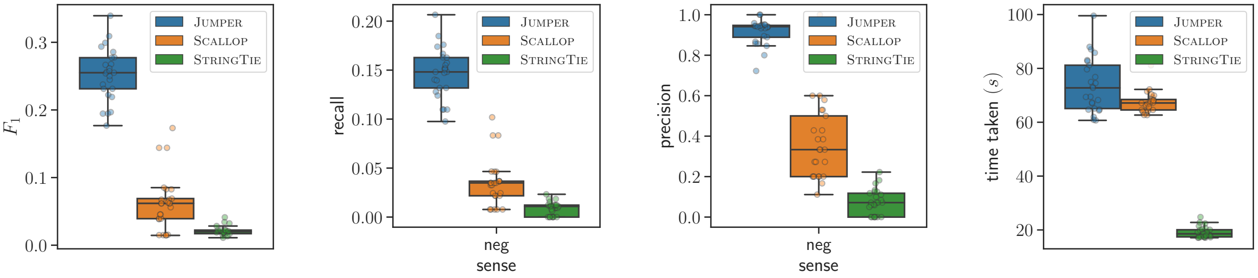

(a) **(b)** **(c)** **(d)**

Figure 5: JUMPER outperforms SCALLOP and STRINGTIE for all simulation instances in terms of F_1 score, recall and precision while maintaining a modest running time. (a) F_1 score (b) recall, (c) precision and (d) time taken by the three methods for the simulated instances.

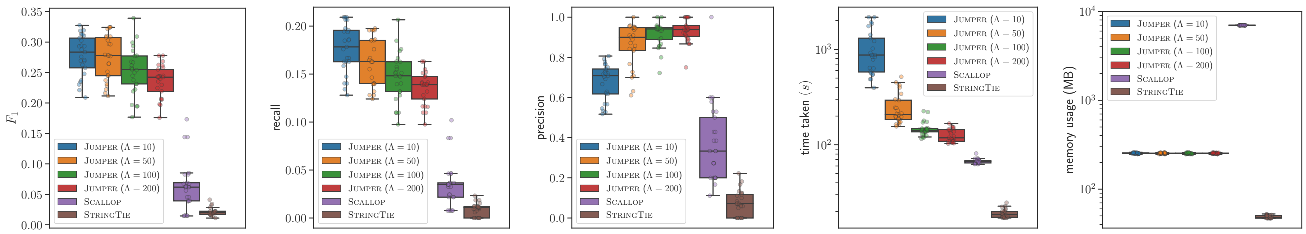

(a) **(b)** **(c)** **(d)** **(e)**

Figure 6: JUMPER outperforms SCALLOP and STRINGTIE for varying values of thresholding parameter Λ. (a) F_1 score (b) recall, (c) precision, (d) single-threaded time taken and (e) memory used by the JUMPER for different values of Λ compared to SCALLOP and STRINGTIE on the simulated instances. As expected, the recall value drops for increasing Λ while the precision increases. We set the default value of Λ to 100 which incurs runtime comparable to SCALLOP while producing higher recall and precision solutions.

- Figure 12 shows the number of *supporting reads* with the 5' end mapping to the leader sequence in the short and long read sequencing data.
- Figure 14 shows the abundances of the predicted transcripts by JUMPER in two SARS-CoV-1 infected samples.
- Table 3 shows summary of the results from the simulations.
- Table 4 describes 18 transcripts (including 9 canonical transcripts) detected from SARS-CoV-2 infected samples with and without pre-treatment of ruxolitinib.

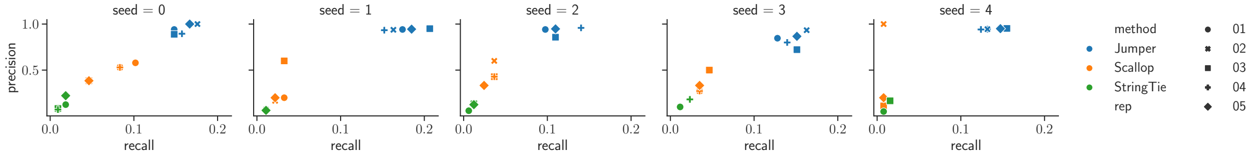

Figure 7: While all three methods return consistent results when generating technical sequencing replicates, JUMPER produces better recall and precision when compared to SCALLOP and STRINGTIE for every simulation instance $(\mathcal{T}, \mathbf{c})$. Varying simulation instances $(\mathcal{T}, \mathbf{c})$ correspond to distinct panels. Each panel shows the recall and precision of the three methods for 5 sequencing experiments of the same simulated instance $(\mathcal{T}, \mathbf{c})$.

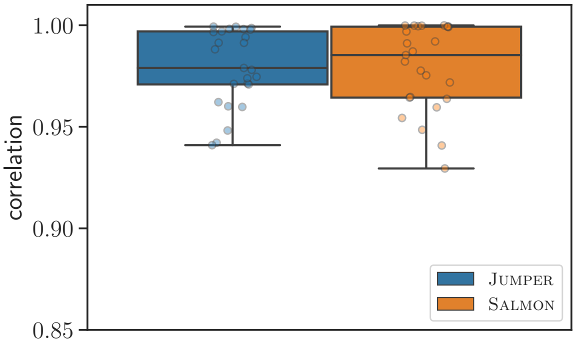

Figure 8: SALMON [15] slightly outperform JUMPER in estimating the abundance of transcripts on simulated instances. This figure shows the Pearson correlation between the true abundances and the abundances estimated by SALMON and JUMPER of transcripts that were identified by JUMPER.

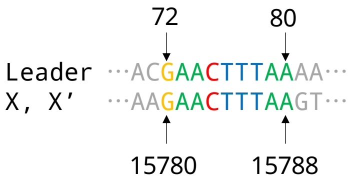

(a) Core sequence

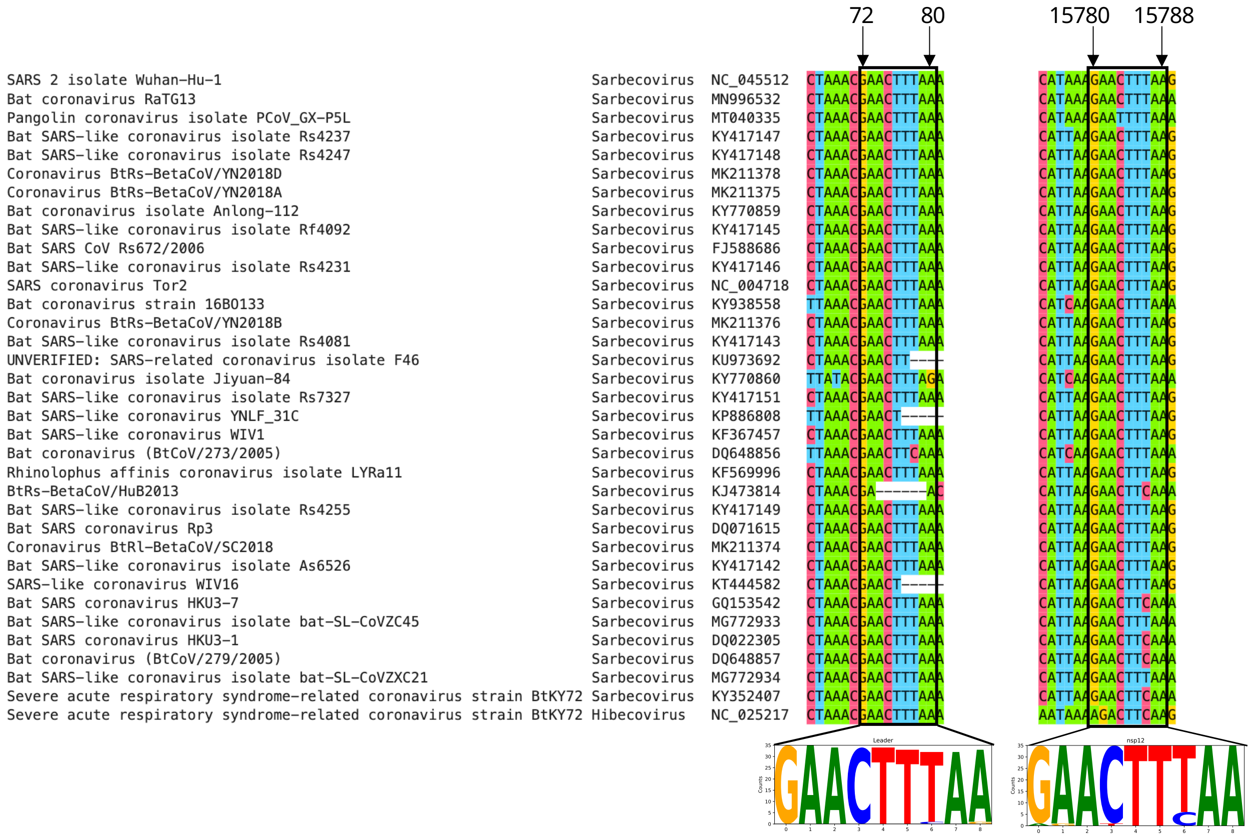

(b) MSA and sequence logo with 34 *Sarbecoviruses* and 1 *Hibecovirus*.

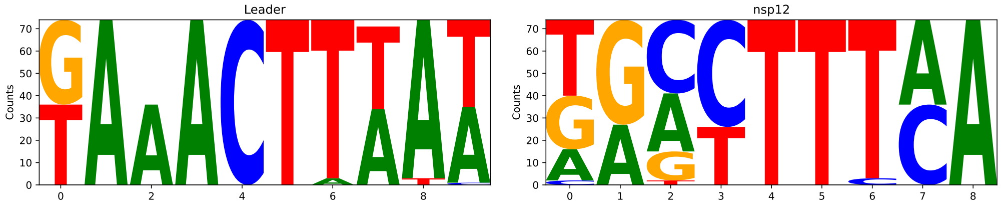

(c) Sequence logo of 11 *Nobecoviruses*, 27 *Merbecoviruses* and 36 *Embecoviruses*

Figure 9: The core sequence of transcript X is conserved within the *Sarbecovirus* subgenus but not in other subgenera of the *Betacoronavirus* genus. (a) Core sequence for the transcript X and X'. (b) Sequence logo for the positions 15780 to 15788 in SARS-CoV-2 genome built from the multiple sequence alignment of the leader sequence and ORF1ab of 34 *Sarbecovirus* and a *Hibecovirus*. (c) Sequence logo for positions 15780 to 15788 in SARS-CoV-2 genome built from multiple sequence alignment with the remaining subgenera of *Betacoronaviruses*.

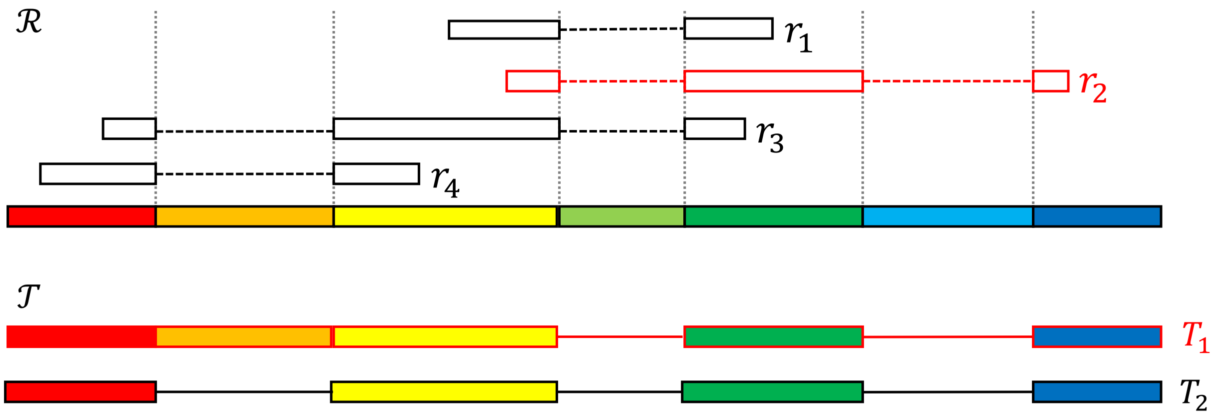

Figure 10: A schematic showing an example of a supporting read for a transcript T_1 with $\sigma^{\oplus}(T_1) = 2$. Transcript T_1 is supported by r_2 because $\pi(r_2) = \pi(T_1)$ and $|\sigma^{\oplus}(r_1)| = |\sigma^{\oplus}(T_1)| = 2$. Reads r_1, r_3 and r_4 do not support T_1 since $|\sigma^{\oplus}(r_1)| < |\sigma^{\oplus}(T_1)|$ and $\pi(r_3), \pi(r_4) \nsubseteq \pi(T_1)$. No reads support T_2 since $|\sigma^{\oplus}(r_j)| < |\sigma^{\oplus}(T_2)|$ for all reads r_j.

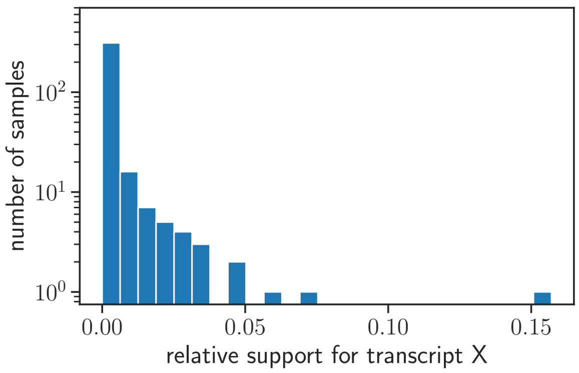

(a) short-read samples

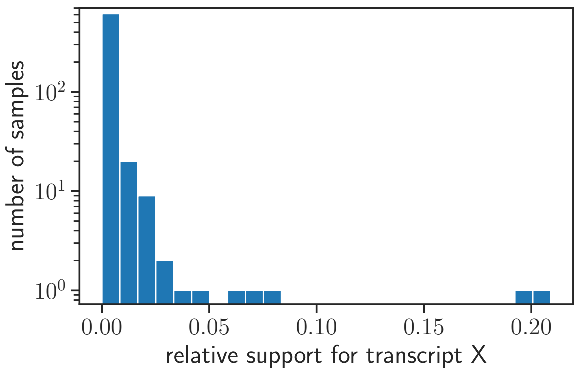

(b) long-read samples

Figure 11: Transcript X has supporting reads in multiple independent publicly available samples of SARS-CoV-2 infected cells on SRA. Distribution of number of (a) short-read and (b) long-read SRA samples with varying proportion of leader-sequence spanning reads that support transcript X. All the short-read samples were aligned using `STAR` [33] while the long-read samples were aligned using `minimap2` [36]. In this plot we only consider samples with more than 100 reads that map to the leader-sequence (position 55 to 85 in the SARS-CoV-2 reference genome).

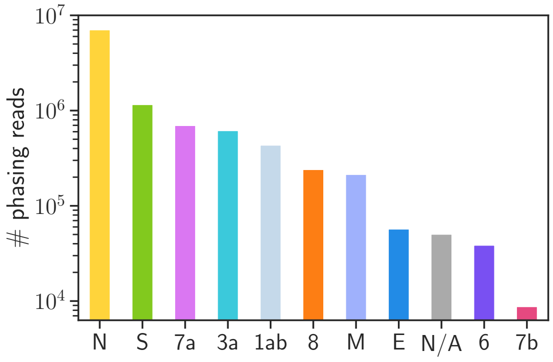

(a)

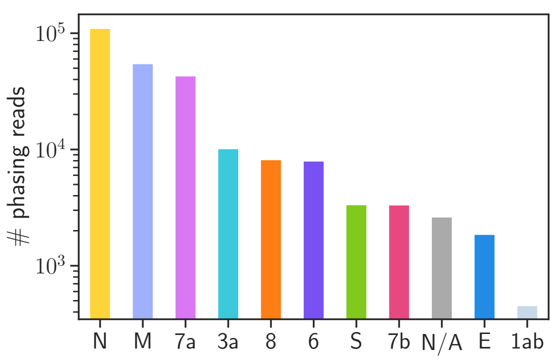

(b)

Figure 12: Supporting phasing reads with 5’ end mapping to the leader sequence in short and long-read sequencing samples of SARS-CoV-2 infected Vero cells [12]. Supporting phasing reads have at most one discontinuous edge with the 5’ end occurring in the leader sequence (*i.e.* between positions 55 and 85) and the first occurrence of ‘AUG’ downstream of the 3’ end position coinciding with the start codon of a known ORF. Supporting phasing reads corresponding to ‘1ab’ start in the leader sequence but do not contain a discontinuous edge. Supporting phasing reads corresponding to ‘N/A’ start in the leader sequence but have a 3’ end such that the first occurrence of ‘AUG’ downstream of the 3’ end position does *not* coincide with the start codon of any known ORFs. (a) Supporting phasing reads in the short-read sequencing sample. (b) Supporting phasing reads in the long-read sequencing sample.

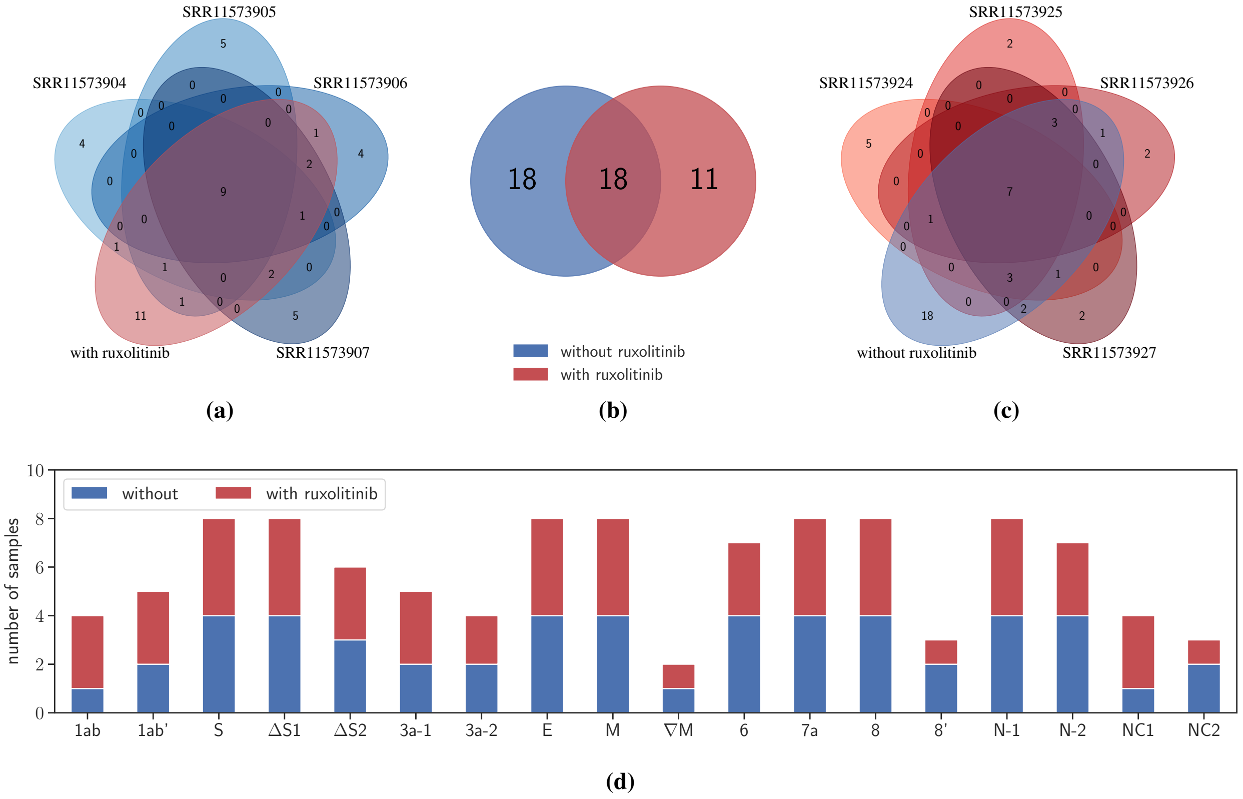

Figure 13: JUMPER enables analysis of drug response of the virus in infected cells at the transcript level.

(a) A Venn diagram of reconstructed transcripts from each of the 4 technical replicate samples (SRR11573904, SRR11573905, SRR11573906 and SRR11573907) without treatment of ruxolitinib (in blue), and all the transcripts from technical replicate samples with treatment pooled together (in red). (b) A Venn diagram showing the number of transcripts reconstructed from samples with and samples without treatment with ruxolitinib (*i.e.*, two groups of four technical replicates). (c) A Venn diagram of reconstructed transcripts from each of the 4 technical replicate samples (SRR11573924, SRR11573925, SRR11573926 and SRR11573927) with treatment of ruxolitinib (in red), and all the transcripts from technical replicate samples without treatment of ruxolitinib pooled together (in blue). (d) A bar plot showing the number of samples containing each of the 18 common transcripts reconstructed from samples both with and without treatment of ruxolitinib. Table 4 described each of the 18 common transcripts. The transcripts are named based on the protein they yield, with ∇ indicating presence of out-of-frame deletions and Δ indicating in-frame deletions.

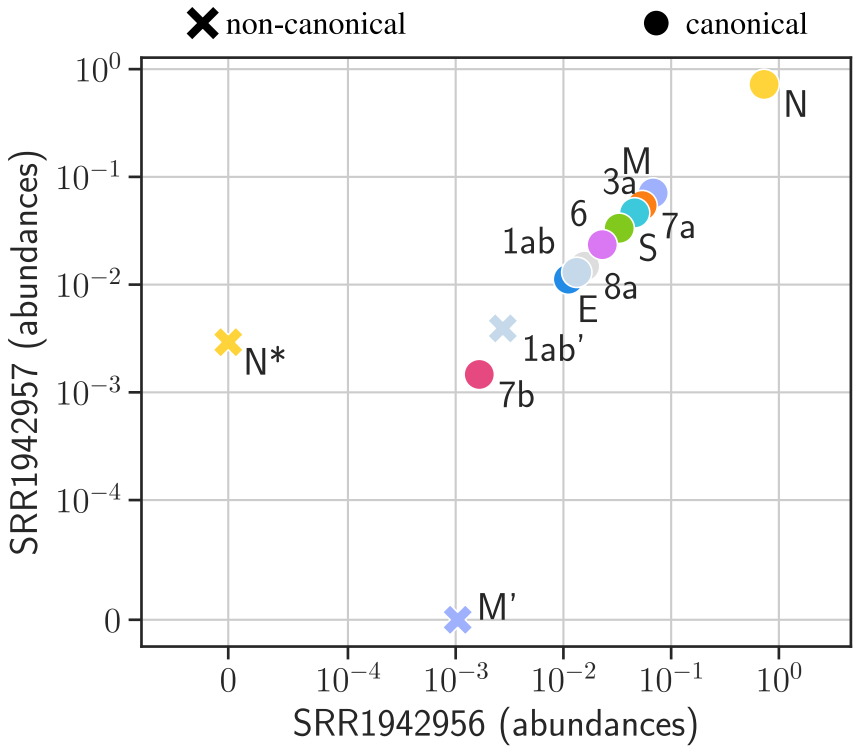

Figure 14: Abundances of the canonical and non-canonical transcripts predicted by JUMPER are consistent in the two SARS-CoV-1 infected samples (SRR194256 and SRR194257). JUMPER predicts 10 canonical and 3 non-canonical transcripts across the two samples.

Simulation				JUMPER			SCALLOP			STRINGTIE		
seed	rep	can	non-can	TP		FP	TP		FP	TP		FP
				can	non-can		can	non-can		can	non-can	
0	1	14	94	7	9	1	7	4	8	2	0	14
0	2	14	94	8	11	0	7	2	8	1	0	13
0	3	14	94	7	11	2	4	1	8	1	0	11
0	4	14	94	6	9	2	7	2	8	1	0	13
0	5	14	94	7	11	0	4	1	8	1	0	7
1	1	14	78	3	13	1	3	0	12	2	0	13
1	2	14	78	4	11	1	2	0	10	1	0	13
1	3	14	78	3	16	1	3	0	2	1	0	12
1	4	14	78	3	11	1	2	0	8	0	0	16
1	5	14	78	4	13	1	2	0	8	1	0	15
2	1	14	150	5	11	1	3	1	8	1	0	16
2	2	14	150	4	14	3	5	1	4	2	0	15
2	3	14	150	5	13	3	5	1	8	2	0	13
2	4	14	150	7	16	1	5	1	8	2	0	16
2	5	14	150	4	14	1	3	1	8	2	0	14
3	1	14	72	4	7	2	3	0	8	1	0	9
3	2	14	72	6	8	2	3	0	4	0	0	8
3	3	14	72	7	6	4	4	0	8	0	0	20
3	4	14	72	4	8	3	3	0	8	2	0	9
3	5	14	72	4	9	2	3	0	6	0	0	4
4	1	14	115	4	13	1	1	0	4	1	0	19
4	2	14	115	5	12	1	1	0	0	0	0	12
4	3	14	115	6	14	1	1	0	8	2	0	10
4	4	14	115	6	10	1	1	0	4	0	0	16
4	5	14	115	6	13	1	1	0	4	0	0	12

Table 3: Simulation results for the three methods JUMPER, SCALLOP and STRINGTIE. Each distinct value in the column 'seed' is a unique instance of $(\mathcal{T}, \mathbf{c})$ and each distinct value in the column 'rep' is a unique sequencing experiment for the given $(\mathcal{T}, \mathbf{c})$. (rep: replicate, can: canonical, non-can: non-canonical, TP: true positives, FP: false positives)

Transcript	Discontinuous Edges	Description
1ab	-	canonical transcript with no discontinuous edges
1ab’	(23593, 23630)	single discontinuous edge downstream of ORF1ab
S	(65, 21552)	single discontinuous edge from TRS-L to TRS-B of ORF S
ΔS1	(65, 21552)<br>(23593, 23630)	single discontinuous edge from TRS-L to TRS-B of ORF S<br>and an in-frame 12 amino-acid deletion overlapping the furin cleavage site
ΔS1	(65, 21552)<br>(23593, 23615)	single discontinuous edge from TRS-L to TRS-B of ORF S<br>and an in-frame 7 amino-acid deletion overlapping the furin cleavage site
3a-1	(65, 25381)	single discontinuous edge from TRS-L to TRS-B of ORF3a
3a-2	(66, 27385)	single discontinuous edge from TRS-L to TRS-B of ORF3a
E	(69, 26237)	single discontinuous edge from TRS-L to TRS-B of ORF E
M	(64, 26468)	single discontinuous edge from TRS-L to TRS-B of ORF M
∇M	(64, 26468)<br>(26779, 26817)<br>(28525, 28577)	single discontinuous edge from TRS-L to TRS-B of ORF M<br>with an out of frame deletion with motifs ‘CAATGGCTT’ to ‘CATTGCTT’<br>and another downstream deletion within ORF N
6	(69, 27041)	single discontinuous edge from TRS-L to TRS-B of ORF6
7a	(66, 27385)	single discontinuous edge from TRS-L to TRS-B of ORF7a
8	(65, 27884)	single discontinuous edge from TRS-L to TRS-B of ORF8
8’	(65, 27884)<br>(28270, 28970)	single discontinuous edge from TRS-L to TRS-B of ORF8<br>with a single deletion downstream of ORF8
N-1	(64, 28255)	single discontinuous edge from TRS-L to TRS-B of ORF N
N-2	(68, 28263)	single discontinuous edge from TRS-L to TRS-B of ORF N
NC1	(6001, 27376)	matching motif ‘AGAGCAACCAAT’ on the 5’ and 3’ ends of the jump
NC2	(731, 29307)	matching motif ‘ATTTTCAA’ to ‘AATTTCAA’

Table 4: 18 transcripts (including 9 canonical transcripts) detected from SARS-CoV-2 infected A549 cell line samples with and without pre-treatment of ruxolitinib. Figure 5 in the main text shows the abundances of these transcripts in the samples.

References

[1] Cole Trapnell, Brian A Williams, Geo Pertea, Ali Mortazavi, Gordon Kwan, Marijke J Van Baren, Steven L Salzberg, Barbara J Wold, and Lior Pachter. Transcript assembly and quantification by RNA-Seq reveals unannotated transcripts and isoform switching during cell differentiation. *Nature Biotechnology*, 28(5):511–515, 2010.

[2] Lasse Maretty, Jonas Andreas Sibbesen, and Anders Krogh. Bayesian transcriptome assembly. *Genome biology*, 15(10):1–11, 2014.

[3] Li Song and Liliana Florea. CLASS: constrained transcript assembly of RNA-seq reads. In *BMC Bioinformatics*, volume 14, page S14. Springer, 2013.

[4] Wei Li, Jianxing Feng, and Tao Jiang. Isolasso: a lasso regression approach to rna-seq based transcriptome assembly. *Journal of Computational Biology*, 18(11):1693–1707, 2011.

[5] Mihaela Pertea, Geo M Pertea, Corina M Antonescu, Tsung-Cheng Chang, Joshua T Mendell, and Steven L Salzberg. StringTie enables improved reconstruction of a transcriptome from RNA-seq reads. *Nature Biotechnology*, 33(3):290–295, 2015.

[6] Mingfu Shao and Carl Kingsford. Accurate assembly of transcripts through phase-preserving graph decomposition. *Nature Biotechnology*, 35(12):1167–1169, 2017.

[7] Jonas Behr, Andre Kahles, Yi Zhong, Vipin T Sreedharan, Philipp Drewe, and Gunnar Rätsch. Mitie: Simultaneous rna-seq-based transcript identification and quantification in multiple samples. *Bioinformatics*, 29(20):2529–2538, 2013.

[8] Jin Zhao, Haodi Feng, Daming Zhu, and Yu Lin. Multitrans: an algorithm for path extraction through mixed integer linear programming for transcriptome assembly. *IEEE/ACM Transactions on Computational Biology and Bioinformatics*, 2021.

[9] Zhaleh Safikhani, Mehdi Sadeghi, Hamid Pezeshk, and Changiz Eslahchi. Ssp: An interval integer linear programming for de novo transcriptome assembly and isoform discovery of rna-seq reads. *Genomics*, 102(5-6):507–514, 2013.

[10] Jin Zhao, Haodi Feng, Daming Zhu, Chi Zhang, and Ying Xu. Isotree: A new framework for de novo transcriptome assembly from rna-seq reads. *IEEE/ACM transactions on computational biology and bioinformatics*, 17(3):938–948, 2018.

[11] Juntao Liu, Guojun Li, Zheng Chang, Ting Yu, Bingqiang Liu, Rick McMullen, Pengyin Chen, and Xiuzhen Huang. Binpacker: packing-based de novo transcriptome assembly from rna-seq data. *PLoS computational biology*, 12(2):e1004772, 2016.

[12] Dongwan Kim, Joo-Yeon Lee, Jeong-Sun Yang, Jun Won Kim, V Narry Kim, and Hyeshik Chang. The architecture of SARS-CoV-2 transcriptome. *Cell*, 2020.

[13] Cong Ma, Hongyu Zheng, and Carl Kingsford. Exact transcript quantification over splice graphs. In *20th International Workshop on Algorithms in Bioinformatics (WABI 2020)*. Schloss Dagstuhl-Leibniz-Zentrum für Informatik, 2020.

[14] Bo Li and Colin N Dewey. RSEM: accurate transcript quantification from RNA-Seq data with or without a reference genome. *BMC bioinformatics*, 12(1):323, 2011.

[15] Rob Patro, Geet Duggal, Michael I Love, Rafael A Irizarry, and Carl Kingsford. Salmon provides fast and bias-aware quantification of transcript expression. *Nature methods*, 14(4):417–419, 2017.

[16] Nicolas L Bray, Harold Pimentel, Páll Melsted, and Lior Pachter. Near-optimal probabilistic RNA-seq quantification. *Nature Biotechnology*, 34(5):525–527, 2016.

[17] Laura H Tung, Mingfu Shao, and Carl Kingsford. Quantifying the benefit offered by transcript assembly with Scallop-LR on single-molecule long reads. *Genome Biology*, 20(1):1–18, 2019.

[18] Michael A Quail, Miriam Smith, Paul Coupland, Thomas D Otto, Simon R Harris, Thomas R Connor, Anna Bertoni, Harold P Swerdlow, and Yong Gu. A tale of three next generation sequencing platforms: comparison of ion torrent, pacific biosciences and illumina miseq sequencers. *BMC genomics*, 13(1):1–13, 2012.

[19] Jiawen Cui, Zhaogeng Lu, Guolu Xu, Yuyao Wang, Biao Jin, et al. Analysis and comprehensive comparison of pacbio and nanopore-based rna sequencing of the arabidopsis transcriptome. *Plant Methods*, 16(1):1–13, 2020.

[20] Jason L Weirather, Mariateresa de Cesare, Yunhao Wang, Paolo Piazza, Vittorio Sebastiano, Xiu-Jie Wang, David Buck, and Kin Fai Au. Comprehensive comparison of pacific biosciences and oxford nanopore technologies and their applications to transcriptome analysis. *F1000Research*, 6, 2017.

[21] Shanika L Amarasinghe, Shian Su, Xueyi Dong, Luke Zappia, Matthew E Ritchie, and Quentin Gouil. Opportunities and challenges in long-read sequencing data analysis. *Genome biology*, 21(1):1–16, 2020.

[22] Daniel Blanco-Melo, Benjamin E Nilsson-Payant, Wen-Chun Liu, Skyler Uhl, Daisy Hoagland, Rasmus Møller, Tristan X Jordan, Kohei Oishi, Maryline Panis, David Sachs, et al. Imbalanced host response to SARS-CoV-2 drives development of COVID-19. *Cell*, 2020.

[23] Jon Lee and Dan Wilson. Polyhedral methods for piecewise-linear functions I: the lambda method. *Discrete Applied Mathematics*, 108(3):269–285, 2001.

[24] Alyson Imamoto and Benjamim Tang. A recursive descent algorithm for finding the optimal minimax piecewise linear approximation of convex functions. In *Advances in Electrical and Electronics Engineering-IAENG Special Edition of the World Congress on Engineering and Computer Science 2008*, pages 287–293. IEEE, 2008.

[25] Alexander O Pasternak, Willy JM Spaan, and Eric J Snijder. Nidovirus transcription: how to make sense…? *Journal of General Virology*, 87(6):1403–1421, 2006.

[26] Stanley G Sawicki and Dorothea L Sawicki. Coronaviruses use discontinuous extension for synthesis of subgenome-length negative strands. In *Corona-and Related Viruses*, pages 499–506. Springer, 1995.

[27] Guido Van Marle, Jessika C Dobbe, Alexander P Gultyaev, Willem Luytjes, Willy JM Spaan, and Eric J Snijder. Arterivirus discontinuous mRNA transcription is guided by base pairing between sense and antisense transcription-regulating sequences. *Proceedings of the National Academy of Sciences*, 96(21):12056–12061, 1999.

[28] Sonia Zuniga, Isabel Sola, Sara Alonso, and Luis Enjuanes. Sequence motifs involved in the regulation of discontinuous coronavirus subgenomic RNA synthesis. *Journal of Virology*, 78(2):980–994, 2004.

[29] Alexander O Pasternak, Erwin van den Born, Willy JM Spaan, and Eric J Snijder. Sequence requirements for RNA strand transfer during nidovirus discontinuous subgenomic RNA synthesis. *The EMBO Journal*, 20(24):7220–7228, 2001.

[30] Dorothea L Sawicki, Tao Wang, and Stanley G Sawicki. The RNA structures engaged in replication and transcription of the A59 strain of mouse hepatitis virus. *Journal of General Virology*, 82(2):385–396, 2001.

[31] Antoine AF de Vries, Amy L Glaser, Martin JB Raamsman, and Peter JM Rottier. Recombinant equine arteritis virus as an expression vector. *Virology*, 284(2):259–276, 2001.

[32] Alyssa C Frazee, Andrew E Jaffe, Ben Langmead, and Jeffrey T Leek. Polyester: simulating RNA-seq datasets with differential transcript expression. *Bioinformatics*, 31(17):2778–2784, 2015.

[33] Alexander Dobin, Carrie A Davis, Felix Schlesinger, Jorg Drenkow, Chris Zaleski, Sonali Jha, Philippe Batut, Mark Chaisson, and Thomas R Gingeras. STAR: ultrafast universal RNA-seq aligner. *Bioinformatics*, 29(1):15–21, 2013.

[34] Xi Zhang, Hin Chu, Lei Wen, Huiping Shuai, Dong Yang, Yixin Wang, Yuxin Hou, Zheng Zhu, Shuofeng Yuan, Feifei Yin, et al. Competing endogenous RNA network profiling reveals novel host dependency factors required for MERS-CoV propagation. *Emerging microbes & infections*, 9(1):733–746, 2020.

[35] Yiyan Yang, Wei Yan, A Brantley Hall, and Xiaofang Jiang. Characterizing Transcriptional Regulatory Sequences in Coronaviruses and Their Role in Recombination. *Molecular Biology and Evolution*, 11 2020. msaa281.

[36] Heng Li. Minimap2: pairwise alignment for nucleotide sequences. *Bioinformatics*, 34(18):3094–3100, 2018.
