# Supplementary material for: Jumper enables discontinuous transcript assembly in coronaviruses
Source: Nat Commun. 2021 Nov 18;12:6728. doi: 10.1038/s41467-021-26944-y (PMC8602663; doi:10.1038/s41467-021-26944-y)
Supplement: Supplementary file 2 — Reporting Summary [file 41467_2021_26944_MOESM2_ESM.pdf]

nature portfolio

Corresponding author(s): Mohammed El-Kebir

Last updated by author(s): October 11, 2021

Reporting Summary

Nature Portfolio wishes to improve the reproducibility of the work that we publish. This form provides structure for consistency and transparency in reporting. For further information on Nature Portfolio policies, see our Editorial Policies and the Editorial Policy Checklist.

Statistics

For all statistical analyses, confirm that the following items are present in the figure legend, table legend, main text, or Methods section.

n/a	Confirmed	
☐	☒	The exact sample size (*n*) for each experimental group/condition, given as a discrete number and unit of measurement
☐	☒	A statement on whether measurements were taken from distinct samples or whether the same sample was measured repeatedly
☐	☒	The statistical test(s) used AND whether they are one- or two-sided<br>*Only common tests should be described solely by name; describe more complex techniques in the Methods section.*
☒	☐	A description of all covariates tested
☐	☒	A description of any assumptions or corrections, such as tests of normality and adjustment for multiple comparisons
☐	☒	A full description of the statistical parameters including central tendency (e.g. means) or other basic estimates (e.g. regression coefficient) AND variation (e.g. standard deviation) or associated estimates of uncertainty (e.g. confidence intervals)
☐	☒	For null hypothesis testing, the test statistic (e.g. *F*, *t*, *r*) with confidence intervals, effect sizes, degrees of freedom and *P* value noted<br>*Give P values as exact values whenever suitable.*
☐	☒	For Bayesian analysis, information on the choice of priors and Markov chain Monte Carlo settings
☒	☐	For hierarchical and complex designs, identification of the appropriate level for tests and full reporting of outcomes
☐	☒	Estimates of effect sizes (e.g. Cohen's *d*, Pearson's *r*), indicating how they were calculated

Our web collection on statistics for biologists contains articles on many of the points above.

Software and code

Policy information about availability of computer code

Data collection	No software was used for data collection.
Data analysis	We developed Jumper to reconstruct viral transcripts from short-read paired-end RNA-seq data of infected cells. Jumper is open source (MIT license) and publicly available at https://github.com/elkebir-group/Jumper. Jumper uses Gurobi (v9.0.3) to solve underlying mixed-integer linear program. We used fastp (v0.20.1) to trim the short reads and quality control. Samtools (v1.9) was used for indexing, sorting and computing coverage of BAM files. The alignment was performed for short-read and long-read sequencing samples using STAR (v2.7.5b) and minimap2 (v2.17), respectively. We simulated short-read sequencing data using Polyester (v1.22.0). Reading of bam files was performed using Pysam (v0.15.3). Transcript abundance estimation was performed using Salmon (v1.4.0). We benchmarked Jumper against Scallop (v0.10.4) and StringTie (v2.1.4). Python v3.6.12 was used for running Jumper as well as analyses and plotting using pandas (v1.1.3), matplotlib (v3.3.2) and seaborn (v0.11.1).

For manuscripts utilizing custom algorithms or software that are central to the research but not yet described in published literature, software must be made available to editors and reviewers. We strongly encourage code deposition in a community repository (e.g. GitHub). See the Nature Portfolio guidelines for submitting code & software for further information.

Data

Policy information about availability of data

All manuscripts must include a data availability statement. This statement should provide the following information, where applicable:

- Accession codes, unique identifiers, or web links for publicly available datasets
- A description of any restrictions on data availability
- For clinical datasets or third party data, please ensure that the statement adheres to our policy

We analyzed previously published datasets in our study. The sequencing data deposited into the Open Science Framework (OSF) at https://doi.org/10.17605/OSF.IO/8F6N9 was analyzed. We also employed our method, Jumper, on the RNA-seq data available on SRA database with the following accession numbers -- SRR11573904, SRR11573905, SRR11573906, SRR11573907, SRR11573924, SRR11573925, SRR11573926, SRR11573927, SRR1942956, SRR1942957, SRR10357372, SRR10357373 and SRR10357374. Simulated data generated in our study is available at https://databank.illinois.edu/datasets/IDB-6667667. Processed data and results of all methods are available at https://github.com/elkebir-group/Jumper-data. Jumper software has been archived on Zenodo, and is available at https://zenodo.org/badge/latestdoi/309318448.

Field-specific reporting

Please select the one below that is the best fit for your research. If you are not sure, read the appropriate sections before making your selection.

- [x] Life sciences
- [ ] Behavioural & social sciences
- [ ] Ecological, evolutionary & environmental sciences

For a reference copy of the document with all sections, see nature.com/documents/nr-reporting-summary-flat.pdf

Life sciences study design

All studies must disclose on these points even when the disclosure is negative.

Sample size	No experimental data was generated in this study. Here, we analyzed previously published datasets in our study. Specifically, we analyzed all SARS-CoV-2 long and short-read sequencing data from (Kim et al., Cell 2020). In addition, we analyzed two biological A549 cell line samples (four technical replicates each, one sample with Ruxolitinib treatment and one without) from (Blanco et al., Cell 2020) that were sequenced 24h after infection with SARS-CoV-2 (MOI 2). From (Zhang et al., Emerging microbes & infection 2020), we analyzed three MERS-CoV infected Calu-3 RNA-seq samples (24 hours post infection) and two SARS-CoV-1 RNA-seq samples (24 hours post infection). To assess the presence of transcript X in SARS-CoV-2, we analyzed a total of 10,047 next-generation sequencing samples (where 4634 are short-read sequencing and 5413 are long-read sequencing samples) of SARS-CoV-2 from the Sequence Read Archive with a date cut-off of 15th June 2020 that had more than 100 reads that map to the leader sequence (position 55 to 85 in the genome).
Data exclusions	To analyze presence the of transcript X, we excluded samples that had low SARS-CoV-2 coverage and only considered samples in which more than 100 reads mapped to the leader sequence (position 55 to 85 in the genome).
Replication	No biological experiments were conducted. The simulated data is available at https://databank.illinois.edu/datasets/IDB-6667667. We also provide a snakemake file for the entire pipeline used in our study.
Randomization	Randomization is not relevant to our study, as no experimental data was generated.
Blinding	Blinding is not relevant to our study, as no experimental data was generated.

Reporting for specific materials, systems and methods

We require information from authors about some types of materials, experimental systems and methods used in many studies. Here, indicate whether each material, system or method listed is relevant to your study. If you are not sure if a list item applies to your research, read the appropriate section before selecting a response.

Materials & experimental systems

n/a	Involved in the study
☒	☐ Antibodies
☒	☐ Eukaryotic cell lines
☒	☐ Palaeontology and archaeology
☒	☐ Animals and other organisms
☒	☐ Human research participants
☒	☐ Clinical data
☒	☐ Dual use research of concern

Methods

n/a	Involved in the study
☒	☐ ChIP-seq
☒	☐ Flow cytometry
☒	☐ MRI-based neuroimaging
